# Extrusion fountains are restricted by WAPL-dependent cohesin release and CTCF barriers

**DOI:** 10.1093/nar/gkaf549

**Published:** 2025-06-30

**Authors:** Ning Qing Liu, Mikhail Magnitov, Marijne M G A Schijns, Tom van Schaik, Hans Teunissen, Bas van Steensel, Elzo de Wit

**Affiliations:** Division of Gene Regulation, The Netherlands Cancer Institute, 1066CX Amsterdam, the Netherlands; Department of Hematology, Erasmus Medical Center Cancer Institute, 3015GD Rotterdam, the Netherlands; Division of Gene Regulation, The Netherlands Cancer Institute, 1066CX Amsterdam, the Netherlands; Division of Gene Regulation, The Netherlands Cancer Institute, 1066CX Amsterdam, the Netherlands; Division of Gene Regulation, The Netherlands Cancer Institute, 1066CX Amsterdam, the Netherlands; Division of Gene Regulation, The Netherlands Cancer Institute, 1066CX Amsterdam, the Netherlands; Division of Gene Regulation, The Netherlands Cancer Institute, 1066CX Amsterdam, the Netherlands; Division of Molecular Genetics, Oncode Institute, The Netherlands Cancer Institute, 1066CX Amsterdam, the Netherlands; Division of Gene Regulation, The Netherlands Cancer Institute, 1066CX Amsterdam, the Netherlands

## Abstract

Interphase chromosomes are mainly shaped by loop extrusion and compartmentalisation mechanisms. However, their temporal component and cause-effect relationships remain largely unknown. In this study, we use acute degradation of WAPL, CTCF and cohesin in mouse embryonic stem cells to investigate the dynamics of loop extrusion and its relationship to compartmentalisation. Stabilisation of cohesin on chromatin by depletion of WAPL results in the formation of extended loops and promotes looping between non-convergent CTCF sites. Loss of WAPL also results in a rapid decrease in compartmentalisation, which is reversed by subsequent removal of cohesin, directly demonstrating the opposite role of extrusion on compartmentalisation. Using combined depletion of WAPL and CTCF, we identify fountains, a feature of chromosome organisation that emanates from enhancer regions and exhibits strong cohesin binding. Fountains form rapidly after mitosis and early in mammalian development. Cohesin depletion confirms that fountains are cohesin dependent, and their disruption leads to the downregulation of fountain-proximal genes, suggesting a role in gene regulation. Taken together, by exploiting the temporal precision of acute protein depletion, our study reveals fountains as an extrusion-mediated, fast-forming feature of 3D genome organisation.

## Graphical Abstract

**Graphical Abstract:**
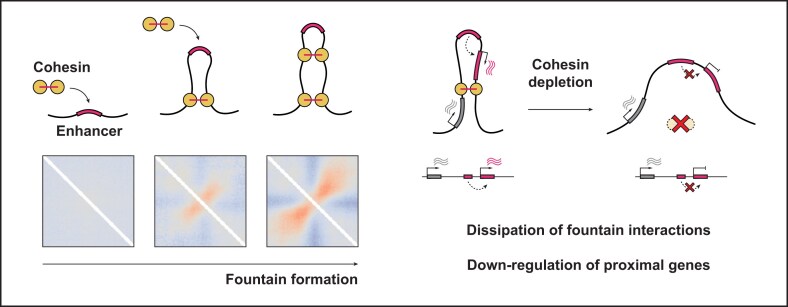

## Introduction

Higher-order genome organisation facilitates fundamental nuclear processes, such as DNA replication, DNA repair and gene expression. Within the nucleus, chromosomes occupy their own specific volumes known as chromosome territories [1]. Each individual chromosome is further segregated into active (A) and inactive (B) compartments [2–4]. Active A compartments are enriched for transcribed genes and active histone modifications such as H3K4me3 and H3K27ac. On the other hand, B compartments tend to be gene-poor and are enriched for histone marks associated with transcriptional repression such as H3K9me3, and are located close to the nuclear periphery [5]. Compartments are generally subdivided into multiple self-interacting regions called topologically associating domains (TADs), which represent a collection of transiently formed chromatin loops [6–8]. In recent years, the mechanisms underlying different levels of genome organisation have been extensively studied and the molecular roles of key regulatory proteins have been elucidated.

The cohesin complex has emerged as one of the major players in interphase genome organisation. Cohesin is a ring-shaped protein complex consisting of the subunits SMC1, SMC3, RAD21 (also called SCC1), and either STAG1 or STAG2 (also known as SA1 or SA2) [9]. This complex is able to bring two distal regions into close proximity to create chromatin loops, which are dominantly formed between two convergently oriented CTCF binding sites [10, 11]. The formation of these loops can be disrupted by deletion or inversion of one of the paired CTCF motifs, indicating the functional importance of CTCF convergence in loop formation [12–14]. Additionally, acute depletion of CTCF or cohesin results in the loss of most chromatin loops [15–18]. Thus, formation of chromatin loops can be explained by the cohesin-mediated loop extrusion model with CTCF acting as a barrier [14, 19].

Single-molecule imaging experiments have shown that human cohesin can extrude DNA *in vitro* [20, 21]. Loop extrusion is considered a highly dynamic process consisting of three major steps: loading of cohesin onto chromatin, extrusion of the chromatin fibre to form loops, and release of cohesin from chromatin in order to reinitiate the extrusion process [22]. Loading of cohesin is promoted by the cohesin loading complex consisting of NIPBL and MAU2 (also called SCC2 and SCC4) and release of cohesin from chromatin is catalysed by WAPL. Knock-out or depletion of the NIPBL or MAU2 leads to a genome-wide loss of chromatin loops due to a failure to load cohesin [23–25], whereas loss of WAPL leads to an increase in loop size as a result of stabilisation of cohesin on DNA [16, 24, 26]. In addition, loss of NIPBL/MAU2 and WAPL leads to an increase and loss of compartmentalisation, respectively [23, 24], suggesting that loop extrusion actively counteracts the compartmentalisation [27].

Although previous research using gene knockouts has provided valuable insights into the central players and mechanisms underlying chromatin organisation, recent work has highlighted the need to study changes in genome structure at a high temporal resolution to separate direct from indirect effects and relate chromatin organisation to other nuclear processes [22, 25, 26, 28–31]. In this work, we aimed to characterise the dynamics of chromatin organisation changes induced by rapid and acute depletion of the key 3D genome regulators WAPL, CTCF and cohesin in mouse embryonic stem cells (mESCs). We show that depletion of WAPL leads to cohesin accumulation at CTCF binding sites, specifically at those that form chromatin loops. Furthermore, stabilisation of cohesin creates illegal loops between CTCF sites, supporting the cohesin traffic jam model [32]. Depletion of both WAPL and WAPL/CTCF leads to a rapid decrease in compartmentalisation, which can be reversed by sequential depletion of cohesin, directly demonstrating that compartmentalisation is counteracted by loop extrusion. In addition, we describe fountains, a type of chromatin structure that emerges from enhancer regions. Fountains are constrained and regulated in size by the combined activity of WAPL and CTCF. We show that fountains are dependent on cohesin-mediated loop extrusion and postulate that these regions may serve as preferential cohesin loading sites on chromatin. Our experiments highlight the power of acute protein depletion in the study of chromosome organisation, allowing us to evaluate existing models and identify novel features of genome structure.

## Materials and methods

### Cell lines

E14Tg2a (129/Ola isogenic background) and the degron tagged cell lines were cultured on 0.1% gelatin-coated plates in serum-free DMEM/F12 (Gibco) and Neurobasal (Gibco) medium (1:1) supplemented with N-2 (Gibco), B-27 (Gibco), BSA (0.05%, Gibco), 104 U of Leukemia Inhibitory Factor/LIF (Millipore), MEK inhibitor PD0325901 (1 μM, Selleckchem), GSK3-β inhibitor CHIR99021 (3 μM, Cayman Chemical) and 1-Thioglycerol (1.5 × 10–4 M, Sigma-Aldrich). The cell lines were passaged every 2 days in daily culture. During the protein depletion experiments, the cells were seeded overnight before the start of the time course in the following densities: For a 96 h time course, 35k, 150k, and 400k cells were seeded in 6-well, 10-cm and 15-cm plates, respectively. For 24 or 27 h time course, 0.5 M, and 4M cells were seeded in 6-well and 15-cm plates, respectively. The medium was refreshed or the cells were split in 1:10 every 2 days during a time course.

#### Acute protein degradation and inhibition

The WAPL and WAPL/CTCF proteins were depleted by treating the cells with a final concentration of 500 μM IAA (Sigma-Aldrich). The RAD21 protein was depleted by adding a final concentration of 500 nM dTAG-13 molecule (Merck Millipore). All the time series experiments were performed by inducing protein degradation at different time points and harvesting the samples at the end of the time course.

#### Plasmid construction

The donor plasmid used to target the endogenous mouse CTCF and RAD21 protein was constructed by modifying a published pEN84 plasmid (Addgene #86 230). To construct the Ctcf donor plasmid, two homology arms around the stop codon of the Ctcf genes, a backbone and an AID peptide were co-amplified by PCR from the pEN84 plasmid. A mCherry fluorescent protein sequence and a hygromycin resistant protein sequence were co-amplified from an in-house available plasmid (Mouse WAPL-AID-mCherry-HygroR donor plasmid). The amplified DNA fragments were joined into a mouse CTCF-AID-mCherry-HygroR donor plasmid using the NEBuilder® HiFi DNA Assembly Cloning Kit (NEB). To construct the Rad21 donor plasmid, the HA-P2A sequence was first removed from the pAW63 (Addgene #104 371). Subsequently, we amplified two homology arms around the C-terminus of the RAD21 gene and the FKBP-BFP sequence and the backbone from the modified pAW63 plasmid. The PCR products were then assembled using the NEBuilder® HiFi DNA Assembly Cloning Kit (NEB). The donor sequences and sgRNAs in the obtained plasmids were validated by Sanger sequencing before using for further experiments.

#### Gene targeting

Generation of the WAPL/CTCF-AID cell line was performed in the background of our WAPL-AID cell line [26]. The donor plasmids and their corresponding sgRNAs (pX335-EN475 and pX335-EN477, Addgene #86 231 and #86 232) for Ctcf targeting were co-transfected into the parental cell lines using Lipofectamine 3000 Reagent (Thermo Fisher). Three days after transfection, the eGFP/mCherry double positive cells were sorted into a gelatinised 96-well plate for single clone selection. The obtained clones were genotyped by PCR and the fusion sequences were validated by Sanger sequencing. The WAPL/CTCF-AID, RAD21-FKBP cell line was further generated in the background of the WAPL/CTCF-AID cell line. For Rad21 targeting, the donor plasmid and the sgRNA (pX330-EN1082, Addgene #156 450) were electroporated into the WAPL/CTCF-AID cells using the Neon Transfection System (Thermo Fisher). We sorted eGFP/mCherry/BFP triple positive cells and performed single clone selection and validation following the same strategy as described above.

#### Western blots

We extracted a nuclear soluble fraction of the mESCs for blotting the WAPL, CTCF, POLR2A and ACTB proteins. For RAD21 and HSP90, mESCs were harvested and lysed in RIPA lysis buffer (150 mM NaCl, 1% NP-40, 0.5% sodium deoxycholate, 0.1% SDS, and 25 mM Tris (pH = 7.4)). The NuPAGETM 4–12% Bis-Tris Protein Gels (Thermo Fisher) were used to separate the proteins. The separated protein was transferred to a pre-activated PVDF membrane using the Trans-Blot Turbo Transfer System (Bio-Rad). The blots were incubated with the following primary antibodies overnight at 4°C: (1) WAPL (1:1000, 16370–1-AP, Proteintech), (2) RAD21 (1:1000, ab154769, Abcam), (3) CTCF (1:1000, 07–729, Merck Millipore), (4) POLR2A (1:2000, 39 097, Active Motif), (5) ACTB (1:5000, AC-15, Abcam) and (6) HSP90 (1:2000, 13171–1-AP). After incubation, the blots were washed 3 times with TBS-0.1% Tween-20. The blots were then incubated with a secondary antibody against rabbit IgG at room temperature for 1 h, followed by 3-time TBS-0.1% Tween-20 washing. The proteins attached with antibodies were hybridised with ClarityTM Western ECL Substrate or Clarity Max Western ECL Substrate reagent (Bio-Rad) and visualised in a ChemiDoc MP Imaging System (Bio-Rad).

#### Flow cytometry

Cell cycle analysis was performed following the protocol of the Click-iT EdU Alexa Fluor 647 Flow Cytometry Assay kit (Invitrogen). For labelling, 10 μM EdU was added to cells, for 1.5 h and incubated at 37°C. After this time cells were harvested, fixed, and permeabilised using a saponin-based permeabilisation and wash reagents provided from the manufacturer. To detect EdU, cells were incubated with Click-iT reaction cocktail for 30 min. For DNA content measure, DAPI (1:1000) was added. EdU and DAPI fluorescent signals were quantified on a BD FACSymphony analyser (BD Biosciences) with the R(C) 670/30 and V(F) 450/50 laser settings. Subsequent analysis was done with FlowJo v10.10.0 software. Single cells were gated, and G1, S and G2 cells were separated and quantified using the indicated gates.

#### ChIP-seq

The ChIP-seq experiments were performed in presence of 10% HEK293T cells as an internal reference using a published protocol with small modifications [33]. For chromatin preparation, the mESCs were mixed with 10% HEK293T cells and cross-linked by a final concentration of 1% formaldehyde for 10 min. The cross-linking reaction was quenched using 2.0 M glycine. The cross-linked cells were then lysed and sonicated to obtain ∼300 bp chromatin using Bioruptor Plus sonication device (Diagenode). For ChIP assays, antibodies were first coupled with Protein G beads (Thermo Fisher), and then the sonicated chromatin was incubated overnight at 4°C with the antibody coupled Protein G beads. After over incubation, captured chromatin was washed, eluted and de-crosslinked. The released DNA fragments were purified using MiniElute PCR Purification Kit (Qiagen). The ChIP experiments were performed using the following antibodies: (1) WAPL (16370–1-AP, Proteintech), (2) CTCF (07–729, Merck Millipore), (3) RAD21 (ab154769, Abcam), and (4) H3K4me1 (pAb-037–050, Diagenode). The purified DNA fragments were prepared according to the protocol of KAPA HTP Library Preparation Kit (Roche) prior to sequencing. All the ChIP-seq libraries were sequenced using the single- end 65-cycle mode on an Illumina HiSeq 2500.

#### ATAC-seq

ATAC-seq experiment was performed following a published protocol [33]. We first isolated the nuclei from the harvested mESCs, and then the nuclei were permeabilised and tagmented using in-house-generated Tn5 transposase. The tagmented DNA was amplified by two sequential nine-cycle PCR amplifications, and the DNA fragments between 150 and 700 bp in size were purified with AMPure XP SPRI beads (Beckman). The ATAC-seq library was sequenced on a NextSeq 550 platform.

#### RNA-seq

RNA was isolated following a standard TRIzol RNA isolation protocol. The cells were lysed using 1 ml of TRIzol LS Reagent (Thermo Fisher), and 200 μL chloroform was added to the lysates. The mixture was vortexed and centrifuged at 12 000 g at 4°C for 15 min. Upper phase was homogenised with 0.5 ml of 100% isopropanol, incubated at room temperature for 10 min, and centrifuged at 4°C for 10 min. The resulting RNA pellet was washed with 75% ice-cold ethanol, dried at room temperature for 10 min, and resuspended in RNase-free water. The isolated RNA was treated with DNase using the RNeasy Mini Kit (Qiagen). RNA-seq libraries were prepared using a TruSeq Stranded RNA LT Kit (Illumina). The libraries were sequenced using the same platform as the ChIP-seq libraries.

#### Hi-C

We generated Hi-C data as previously described [8] with minor modifications [24]. For each template, 10 million cells were harvested and crosslinked using 2% formaldehyde. Crosslinked DNA was digested in the nucleus using MboI (NEB), and biotinylated nucleotides were incorporated at the restriction overhangs and joined by blunt-end ligation. The ligated DNA was enriched in a streptavidin pull-down. Hi-C libraries were prepared using a standard end-repair and A-tailing method and sequenced on an Illumina HiSeq X sequencer generating paired-end 150 bp reads.

#### pA-DamID

pA-DamID experiment was performed using our published protocol [34]. Briefly, two million cells of each experimental condition were harvested and kept on ice. One million cells were used to localise pA-Dam to a Lamin B1 antibody (1:100, ab16048, Abcam). The remaining cells were used as Dam-control by adding 0.5 μL free Dam Methyltransferase (NEB) during incubation with methyl donor S-adenosylmethionine. Genomic DNA was digested with the m6A-specific DpnI restriction enzyme (NEB) and further processed for high-throughput sequencing. Dam-control and Lamin B1 samples were sequenced on an Illumina HiSeq 2500 with approximately 30 million 65 bp single-end reads per sample.

#### ChIP-seq and ATAC-seq analysis

Calibrated ChIP-seq data were analysed using our published pipeline [26]. Raw sequencing data was mapped to a concatenated reference genome (mm10 and hg19) using Bowtie 2 v2.3.4.1 [35]. The spike-in reference (reads mapped to hg19) was used for the calibration in order to properly quantify the samples (reads mapped to mm10). ATAC-seq data were mapped against the mm10 reference genome using bwa mem v0.7.15-r1140 [36]. The mapped reads with MAPQ < 15, as well as optical PCR duplicates, were discarded using SAMtools. The coverage files were generated using the RPGC method in deepTools v3.0.

Alignment of ChIP-seq and ATAC-seq signals were calculated using the ‘computeMatrix’ function from deepTools v3.0 [37]. The ‘reference-point’ method was used for the alignment. Heatmaps were directly visualised using deepTools.

Peaks were identified using MACS2 v2.1.1 [38]. Peaks with at least 10 reads were kept for further analyses. Fimo v4.11.2 [39] was used to locate CTCF motifs in CTCF ChIP-seq peaks using MA0139.1 PWM from the JASPAR database [40].

#### RNA-seq analysis

Raw RNA-seq data were mapped against the mm10 reference genome using STAR v2.7.9a [41] with GENCODE vM25 gene annotation [42]. Read counts per gene were obtained using the ‘quantMode’ parameter in STAR. Differentially expressed genes were identified using the DESeq2 v1.30.1147 [43]. Low expressed genes were filtered by requiring half of the samples to have gene counts greater than 10. Distances between the samples were calculated based on the VST-transformed gene count matrix using the ‘plotPCA’ function. The ‘nbinomWaldTest’ function with default parameters was used to test contrasts. Gene set enrichment analysis was performed using GSEA v4.3.2 [44] in pre-ranked mode with 10 000 permutations and gene sets from MSigDB v2023.2 [45]. Gene sets with FDR < 0.1 were considered significant. To analyse the relationship between fountains and gene expression, genes were overlapped with 100 kb fountain base flanks using bioframe v0.3.0 [46]. For permutation analysis of the genes in fountain base flanks, gene labels (‘down-regulated’, ‘up-regulated’ or ‘stable’) were randomly permuted (N = 100), and fraction of the genes was calculated to obtain mean, min and max values per gene category. E-P genes were identified using previously published E-P loop annotation from Micro-C data [28]. Genes that had a TSS within 2 kb of the annotated loop anchors were considered as E-P genes. The permutation analysis for E-P genes and other genes in the fountain base flanks was performed in the same way as described above.

#### Hi-C analysis

##### Data processing

Generated Hi-C data were mapped with HiC-Pro v2.9.0 [47], which performs mapping, identification of valid Hi-C pairs, generation of contact matrices and ICE normalisation. Publicly available Hi-C data were mapped using the Open2C distiller-nf pipeline (https://github.com/open2c/distiller-nf) with bwa mem v0.7.17-r1188. Mapped reads were parsed using pairtools v0.3.0 [48] with the ‘walks-policy’ parameter set to ‘mask’ and the ‘max_mismatch_bp’ parameter set to 1, binned into Hi-C contact matrices and iteratively corrected using the ‘balance’ function from cooler v0.8.11 [49]. Genome-wide relative contact probability curves and their derivatives were calculated for the 5 kb resolution Hi-C contact matrices using cooltools v0.5.1 [50].

##### Loops analyses

We used the primary loops identified in a deeply sequenced Hi-C dataset from mESCs [51]. Based on the loop anchors of these primary loops, we identified putative extended loops by recombining 5′ and 3′ loop anchors, with distance limits between 1.5 and 3Mb. Average loops were calculated for 5 kb resolution observed-over-expected Hi-C contact matrices using coolpup.py v0.9.5 [52] with ‘pad’ set to 200 and ‘min-dist’ set to 0. Average TADs were calculated for 20 kb resolution observed-over-expected Hi-C contact matrices using coolpup.py v0.9.5 with options ‘–local’, ‘–rescale’, and ‘–rescale_size’ set to 99 pixels. Expected contact matrices were obtained using the ‘compute-expected’ function from cooltools v0.3.2 with the ‘ignore-diags’ parameter set to 0.

##### RSCs analyses

The annotation of RSCs was taken from [26]. To split the Aggregate Peak Analysis for overlap between the loop anchors and RSCs we used the GenomicRanges package. To determine the overlap of RSCs with loop anchors, we reduced the loop anchors to a single set of non-overlapping regions. This set was used to determine whether an individual RSC overlaps with a loop anchor. Similar to the extended loops, pairwise interactions were created between RSCs. For each RSC, we identified overlaps with CTCF motifs (see ChIP-seq analysis section). We selected only those RCSs that overlap with motifs in a single orientation. We then generated pairwise interactions with only these RSCs. Subsequent analyses were performed in GENOVA [53].

##### Compartment analyses

We calculated the compartment scores for 100 kb resolution Hi-C contact matrices using GENOVA [53]. The orientation for the compartment score for the untreated condition was determined using H3K4me1 ChIP-seq data. The later time points are oriented in such a way to give the highest correlation with the compartment score from the untreated samples. Saddle plots are created with cooltools v0.5.2 using the compartment scores annotation from the untreated condition of each cell line. Compartment strength was calculated as the product of the average saddle plot scores for 25% of the strongest A/A and B/B interaction bins divided by the square of the 25% of the strongest A/B interactions, following the previously described method [23].

##### Fountains annotation

To systematically annotate the fountains in the WAPL/CTCF-AID line, we used a recently published fontanka tool [54].

To create a mask for the Hi-C data scanning, we first annotated open chromatin islands (OCIs) using the ATAC-seq peaks in the untreated condition. For every 100 kb bin, we summed up the scores of the peaks it overlaps with. This gives us a score per bin. If the combined score of the bin and its 4 flanking bins is over 100, we consider the bin to be in a region with a high ATAC score. We set a limit to the maximum size of an OCI at 10 consecutive bins of high ATAC scores, i.e. a maximum stretch of 1Mb. Additionally, we required the flanking 1Mb on both sides to be devoid of bins in a high ATAC region. The annotated OCIs (N = 149) were used to create an average OCI based on the 20 kb resolution observed-over-expected Hi-C contact matrices with 1Mb window using cooltools v0.5.2. Mean aggregate OCIs at 6 and 24 h post-depletion were merged into the mask.

We have then followed the steps described in the original fontanka paper to annotate fountains. Since fountains are most prominent at 6 and 24 h post-depletion, we used a merged dataset of these two time points. First, we extracted the snippets from the Hi-C data at 20 kb resolution with 1 Mb window size using the fontanka ‘slice-windows’ command. Second, the fountain score, fountain score prominence and noise score were calculated for each snippet using the fontanka ‘apply-fountain-mask’ command. Then a set of thresholds and filters was applied according to the procedure of the original fontanka paper.

First, we removed the fountains with weak fountain score prominence using Li's thresholding as in the original study. Second, we removed fountains that were located within 100 kb of the nearest bad bin, defined as bins without any normalisation factor present after the iterative correction with cooler v0.8.5. Third, we retained only the fountains with a positive correlation with the fountain mask. Next, we required the fountains detected in the merged 6 and 24 h datasets to be also detected in individual 6 and 24 h datasets (considering them as individual replicates) with an offset of 60 kb on either side from the fountain. Lastly, we filtered out the top 10% of candidate fountains based on their noise score and bottom 30% of candidate fountains based on their fountain score.

Aggregate analysis of the fountains was performed using the pileup function from cooltools v0.5.2. Fountain interaction strength plots were calculated by averaging the values from the diagonal perpendicular to the main diagonal overlapping with the fountain base and three flanking perpendicular diagonals on either side.

To calculate the enrichment of fountain bases overlaps with ChromHMM states for mESCs [55], a permutation test with circular randomisation (N = 100) from regioneR [56] was used.

#### pA-DamID analysis

The DamID adapter was trimmed from the 65 bp single-end reads using Cutadapt v1.11 [57] and custom scripts. The remaining gDNA was mapped to mm10 with BWA-MEM v0.7.17. Further processing was done with custom R scripts. Reads overlapping GATC fragment ends (indicating successful DpnI digestion) were counted into 100 kb bins and subsequently normalised. These bins were first normalised to 1 million reads and with a pseudocount of 1 a log2-ratio over the Dam-only control was calculated. The average signal between replicates was used for downstream analyses. We used the 100 kb resolution for the downstream comparison with the compartment scores.

## Results

### Establishment of a WAPL/CTCF degron cell line

**Figure 1.**
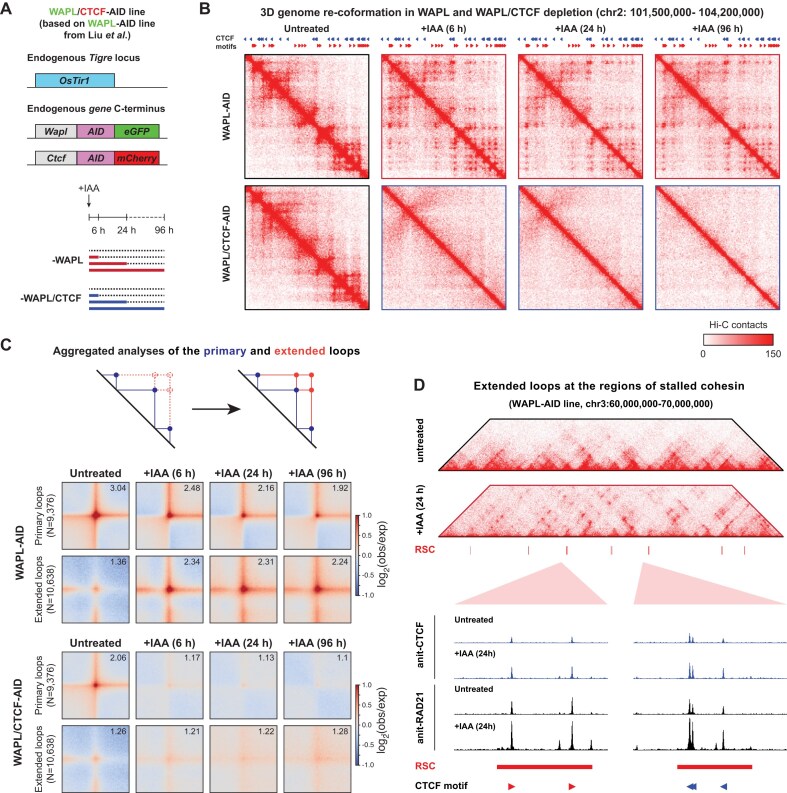
Acute depletion of WAPL or WAPL/CTCF leads to rapid reorganisation of interphase chromosomes. (**A**) In the WAPL-AID cell line [26], the endogenous *Wapl* and *Ctcf* genes were tagged with AID-eGFP and AID-mCherry at the C-terminus of WAPL and CTCF, respectively, to create WAPL/CTCF-AID mESCs (top). Schematic of the depletion conditions: untreated, WAPL, and WAPL/CTCF depletion for 6, 24, and 96 h (bottom). (**B**) An example locus of the Hi-C contact matrices at 20 kb resolution at chromosome 2 showing the rapid formation of the extended loops after WAPL depletion and their dissipation after WAPL/CTCF depletion. CTCF sites are derived from CTCF ChIP-seq data in untreated WAPL-AID cells. Forward and reverse CTCF binding motifs are indicated by triangles. (**C**) Illustrative figure describing the primary and extended loops (top). Aggregate analyses of primary and extended chromatin loops upon WAPL and WAPL/CTCF depletions (bottom). Values in the upper-right corner indicate the interaction strength of the loops over the background. The primary loops and TADs are obtained from [51], and the extended loops are predicted from the primary loop bases using the method described in [24]. (**D**) An example locus of the Hi-C contact matrices at 20 kb resolution showing formation of the extended loops between two regions of stalled cohesin. Zoomed-in regions of ChIP-seq tracks show changes in CTCF and cohesin binding in untreated and WAPL depleted cells.

To study the changes in chromatin organisation upon removal of the strongest regulators of loop extrusion, we generated a double degron mESC line in which both WAPL and CTCF can be acutely depleted. To this end, we modified our previously published WAPL-AID-GFP line [26], which uses the auxin-inducible degron (AID) technology [58], and additionally fused an AID-mCherry to the C-terminus of the endogenous CTCF protein (Fig. 1A). We refer to this double-degron line as the WAPL/CTCF-AID line. Based on immunoblotting, WAPL and CTCF become undetectable within 6 h after the addition of IAA to the culture medium (Supplementary Fig. S1A). Calibrated ChIP-seq following 24 h of IAA treatment showed a clear genome-wide loss of WAPL and CTCF binding as well as RAD21 occupancy at CTCF binding sites in these cells (Supplementary Fig. S1B, C). Importantly, alignment of RAD21 data at the promoters showed that its binding to active TSSs is largely unaffected by the loss of WAPL and CTCF (Supplementary Fig. S1D). Similar to previously published results in non-cycling Wapl^-/-^/Ctcf^-/-^ mouse embryonic fibroblasts, we observed the accumulation of cohesin at the 3′ ends of convergently transcribed genes, forming cohesin islands upon depletion of WAPL and CTCF (Supplementary Fig. S1E) [59]. These results demonstrate that an acute depletion of WAPL and CTCF successfully recapitulated previously published findings based on genetic deletion of the two genes.

We previously showed that both rapid (6 h) and prolonged (96 h) depletion of WAPL does not result in cell cycle defects in mESCs [26]. However, we observed clear growth inhibition of the mESCs after 72 h of WAPL/CTCF co-depletion (Supplementary Fig. S1F). To investigate dynamic changes to the cell cycle following the degradation of both WAPL and CTCF, we performed 5-ethynyl-2′-deoxyuridine (EdU) incorporation experiments. Consistent with our earlier report [60], depletion of WAPL and CTCF for 24 h does not lead to any cell cycle changes (Supplementary Fig. S1G and H). However, after 48 h of depletion, over 60% of the mESCs were arrested in the G1 phase, indicating that prolonged loss of WAPL and CTCF disrupts the cell cycle (Supplementary Fig. S1G and H). These results show that acute depletion of WAPL and CTCF has minimal effects on the proliferation and cell cycle in the first 24 h.

### WAPL prevents extended loop formation between CTCF loop anchors

Our previously published WAPL-AID line and the newly generated WAPL/CTCF-AID line enable us to directly compare what happens to loop extension in the presence and absence of CTCF after cohesin stabilisation. We generated an *in situ* Hi-C dataset with four time points (untreated and 6, 24, and 96 h of IAA treatment) for both degron lines (Fig. 1B). To quantify the overall changes in the 3D genome we performed a relative contact probability (RCP) analysis (Supplementary Fig. S2A). By calculating the derivatives of the RCP curves, it is possible to estimate the average extruded loop size [61]. In the untreated condition the average extruded loop size is 125 and 150 kb in the WAPL-AID and WAPL/CTCF-AID lines, respectively. Upon WAPL depletion the average extruded loop size increases to 380 kb after 6 h and reaches a maximum of 575 and 550 kb at 24 and 96 h post-depletion, respectively. In the absence of both WAPL and CTCF, the average extruded loop size gradually increases with similar steps up to 24 h post-depletion. Interestingly, after 96 h of WAPL and CTCF depletion, the average extruded loop size has further increased to 920 kb (Supplementary Fig. S2A, right panel).

To quantify loop strength at different time points, we used loops identified in a high resolution Hi-C data of the wild-type E14Tg2a mESCs [51], which we refer to as primary loops. Additionally, we generated the pairwise combinations of these primary loop anchors up to 3Mb, which we refer to as the putative extended loops. In the WAPL-AID degron line, we observed clear formation of extended loops as soon as 6 h following IAA treatment (Fig. 1C). When WAPL and CTCF were depleted, both primary and extended loops became undetectable (Fig. 1C). These results show that both WAPL and WAPL/CTCF depletion rapidly change the interaction landscape of chromatin loops and confirm that cohesin positioning at CTCF binding sites is a major mechanism of loop formation in the mammalian genome.

### Stabilisation of cohesin engages CTCF motifs into illegal loops

We have previously shown that stabilisation of cohesin leads to its accumulation at CTCF sites and the formation of extended CTCF-anchored chromatin loops [24, 26]. In mESCs we identified 2789 regions that showed increased cohesin binding, which we termed regions of stalled cohesin (RSCs) [26]. Inspection of our Hi-C maps suggested that loop anchors often overlap with RSCs (Fig. 1D). Indeed, 58% of RSCs (1619 out of 2789) overlap with primary loop anchors [51]. Furthermore, the presence of RSCs at the anchors correlates with emergence of extended loops (Supplementary Fig. S2B, left panel). When anchors overlap with an RSC, the extended loops are strongly induced upon WAPL depletion. In contrast, loop anchors that do not overlap with RSCs form only very weak extended loops (Supplementary Fig. S2B, right panel). Moreover, the locations of the RSCs alone are predictive of the extended loop formation, as shown by the aggregate analysis of the *in silico* loops generated for the pairwise combination of RSCs (Supplementary Fig. S2C). These results indicate that cohesin accumulates at loop anchors and that the amount of this accumulation determines the propensity for looping between two distal loop anchors.

Next, we asked whether the newly formed loops adhered to the CTCF convergence rule. To investigate this, we used RSCs as the proxy for the genomic regions involved in the formation of the extended loops. We stratified the RSCs based on unique CTCF motif orientation and generated a set of putative loops based on the pairwise combination of RSC locations. We observed that the RSCs with convergent CTCF motifs interact most strongly upon WAPL depletion (Supplementary Fig. S2D, top row). However, pairwise combinations between RSCs with tandemly oriented CTCF motifs show an increase of contact frequency that is almost as high as that of the convergently oriented pairs (Supplementary Fig. S2D). These results clearly indicate that loss of WAPL rapidly induces the formation of loops between CTCF sites that do not necessarily follow the convergence rule. Our observations are thus consistent with a model in which the accumulation of cohesin at convergent CTCF sites serves as a roadblock that triggers illegal loop formation for cohesin extruded in the opposite direction [24, 32, 62, 63].

### Chromatin compartmentalisation is uncoupled from nuclear lamina association

**Figure 2.**
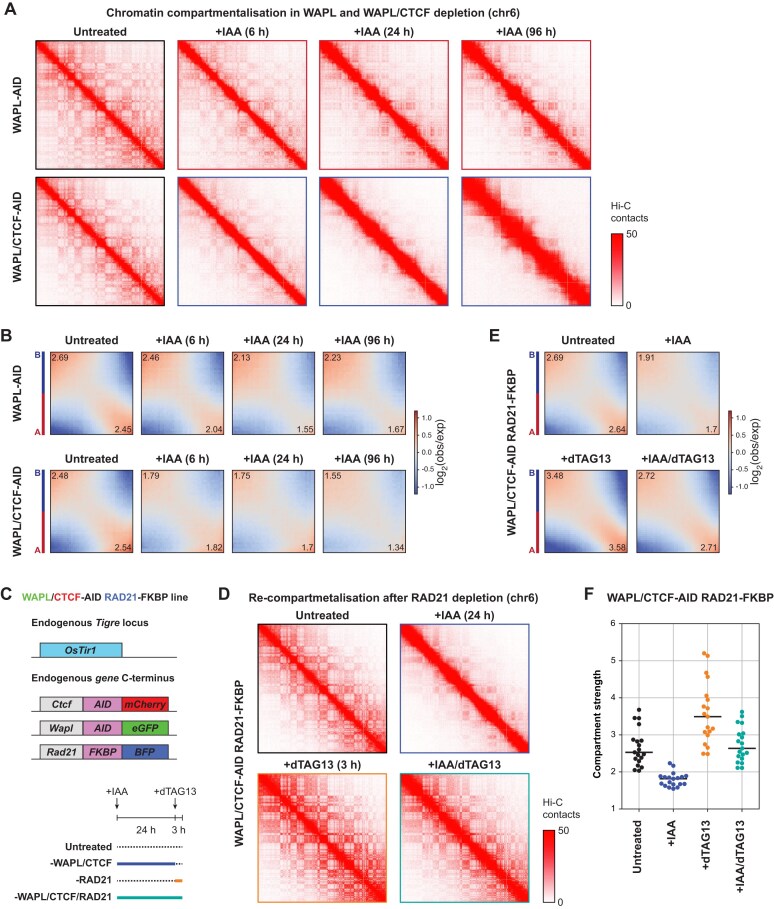
Changes in compartmentalisation are reversed after cohesin depletion. (**A**) An example locus of the Hi-C contact matrices at 100 kb resolution at chromosome 6 showing that WAPL and WAPL/CTCF depletion lead to rapid loss of compartmentalisation in the cells. (**B**) Genome-wide saddle plot analysis for the Hi-C data from the WAPL-AID (top) and WAPL/CTCF-AID (bottom) degron lines following IAA treatment. Saddle plot analysis assesses compartmentalisation, which is defined by higher within-compartment (A/A and B/B) and lower between-compartment (A/B) interactions. Values in the upper-left and bottom-right corners indicate the quantified B and A compartment strengths, respectively [23]. (**C**) In the WAPL/CTCF-AID cell line, the endogenous *Rad21* gene was further fused with a FKBP-BFP sequence at the *C*-terminus of RAD21 (top). Using this cell line, we can sequentially deplete WAPL/CTCF and RAD21 by supplementing first IAA and then dTAG13 in culture medium. Schematic of the sequential depletion conditions: untreated, WAPL/CTCF co-depletion for 24 h, RAD21 depletion for 3 h, WAPL/CTCF co-depletion for 24 h followed by 3 h of RAD21 depletion (bottom). (**D**) Example locus of the Hi-C contact matrices at 100 kb resolution showing Hi-C data from WAPL/CTCF-AID, RAD21-FKBP triple degron line following various degradations. Same region as in (**A**) is shown. (**E**) Genome-wide saddle plot analysis for the Hi-C data from the WAPL/CTCF-AID, RAD21-FKBP degron line following WAPL/CTCF co-depletion for 24 h, RAD21 depletion for 3 h, WAPL/CTCF co-depletion for 24 h followed by 3 h of RAD21 depletion. Values in the upper-left and bottom-right corners indicate the quantified B and A compartment strengths, respectively [23]. (**F**) Genome-wide quantification of compartment strength following sequential depletion in WAPL/CTCF-AID, RAD21-FKBP triple degron line. Each point represents a value for one chromosome. Individual lines indicate the mean values.

Within chromosome territories, active and inactive chromatin segregates into distinct microenvironments called A and B compartments [3, 4]. Compartmentalisation is visible as a checkerboard pattern on Hi-C maps and shows higher than expected homotypic interactions (A/A and B/B) as opposed to heterotypic interactions. Knockout of the cohesin loading factors NIPBL or MAU2 increases compartmentalisation [23, 24], whereas stabilisation of cohesin by disruption of WAPL attenuates it [16, 24]. However, these studies measured A/B compartment segregation after long-term depletion of these cohesin regulators. Using our degron lines, we can assess the direct consequences of losing the key 3D genome regulators on compartmentalisation at high temporal resolution. Following WAPL depletion we find that compartmentalisation is already substantially diminished after 6 h and a maximal reduction after 24 h, with no further reduction at 96 h (Fig. 2A, Supplementary Fig. S3A). Compartmentalisation can be assessed by saddle plots [64], which show that WAPL depletion mainly affects the A/A compartment interactions, whereas B/B compartment interactions are less affected (Fig. 2B, Supplementary Fig. S3A). Simultaneous depletion of WAPL and CTCF also reduces compartmentalisation, but more rapidly, reaching full reduction after 6 h and affecting A and B compartments similarly (Fig. 2B, Supplementary Fig. S3A). These results show that changes in compartmentalisation can vary depending on the presence of CTCF barriers.

B compartments strongly overlap with lamina-associated domains (LADs) and the compartment score shows a strong anti-correlation with the Lamin B1 DamID signal [5]. Our WAPL and WAPL/CTCF degron systems, which enable us to rapidly induce changes in compartmentalisation, are ideal models to investigate the relationship between compartmentalisation and association with the nuclear periphery. To this end, we used the recently developed pA-DamID method [34], which uses antibody-guided *in vitro* Dam methylation and enables taking a snapshot of the genome-lamina interactions. In the untreated cells, there is a strong anti-correlation between the compartment score and the LaminB1 pA-DamID signal (Supplementary Fig. S3B), similar to previous observations [5]. When we compared compartment scores and LaminB1 pA-DamID signal upon WAPL and WAPL/CTCF depletion, we found that despite the massive changes in compartmentalisation, lamina association appears to be largely unaffected (Supplementary Fig. S3B and C). These results demonstrate that nuclear compartmentalisation can be uncoupled from nuclear lamina association, showing that they are not inherently driven by the same molecular mechanism.

### Cohesin depletion rapidly reverses compartmentalisation changes

To confirm that the decrease in compartmentalisation following WAPL depletion is directly driven by the antagonistic process of loop extrusion, we decided to degrade cohesin following the loss of compartmentalisation. To achieve this, we took advantage of another acute depletion strategy called degradation tag (dTAG) [65]. In the WAPL/CTCF-AID cell line, we fused an FKBP^F36V^-BFP to the *C*-terminus of the cohesin subunit RAD21 using our previously published CRISPR-Cas9 strategy (Fig. 2C) [66]. Using this triple degron cell line, we are able to deplete WAPL/CTCF (with IAA) and RAD21 (with dTAG-13) independently, as confirmed by western blot analysis (Supplementary Fig. S4A), suggesting that by combining the degradation systems, we can accurately control the timing and order of WAPL/CTCF and cohesin depletion. Next, we performed *in situ* Hi-C experiments in these triple degron mESCs. Depletion of WAPL/CTCF recapitulated what we had previously found: an increase in contact frequency between loci separated by 1–10 Mb and a loss of loops and TADs (Supplementary Fig. S4B and C). Depletion of RAD21 has previously been shown to result in a loss of CTCF-anchored chromatin loops and TADs [17, 18], which is recapitulated in our triple degron line (Supplementary Fig. S4C). These results show that the triple degron line recapitulates the observations of WAPL/CTCF depletion and previously published RAD21 depletion experiments.

Consistent with the proposed role of loop extrusion in counteracting compartmentalisation, we find that loss of RAD21 alone increases compartment strength (Fig. 2D and E). Next, we analysed compartmentalisation in cells in which we first depleted WAPL/CTCF to reduce A/B compartment segregation, followed by RAD21 depletion. We find that in these cells compartmentalisation is restored to approximately wild-type levels, as indicated by visual inspection of the heatmaps, compartment strength analysis and saddle plot analysis (Fig. 2E and F). These results demonstrate that the cohesin complex is indeed responsible for the restriction of compartmentalisation, most parsimoniously through loop extrusion. Furthermore, it is shown that the restoration of physiological levels of compartmentalisation occurs on the order of hours, indicating that changes in compartmentalisation can be achieved within the span of a single cell cycle.

### WAPL and CTCF constrain the formation of megabase-sized fountains

**Figure 3.**
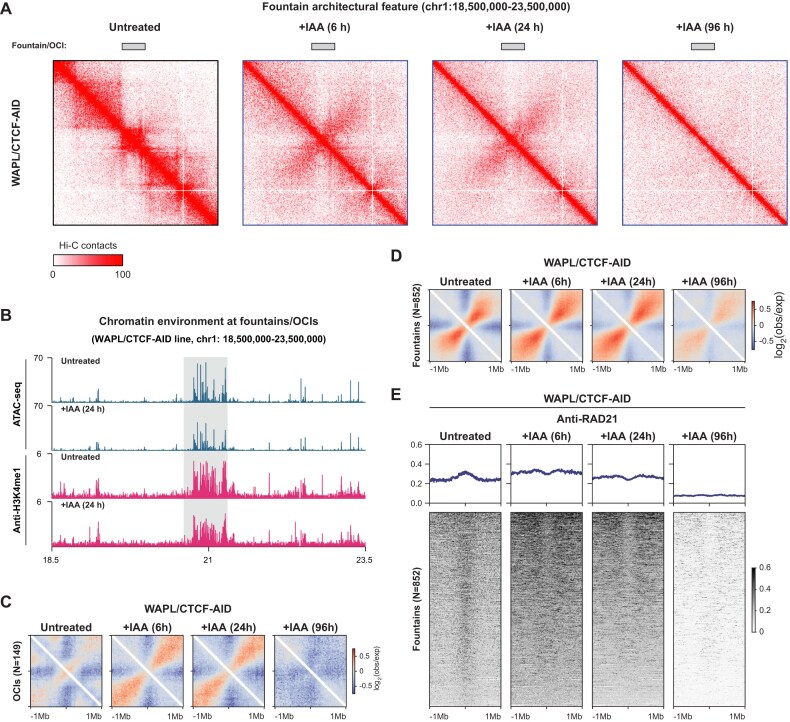
WAPL and CTCF restrict the size of the fountains. (**A**) An example locus of the Hi-C contact matrices at 20 kb resolution at chromosome 1 showing the formation of a fountain after WAPL/CTCF depletion. (**B**) Chromatin environment of a fountain showing accessible chromatin as measured by ATAC-seq and H3K4me1 histone modification ChIP-seq levels in WAPL/CTCF-AID cell line. Rectangle indicates the position of the fountain. (**C**) Aggregate region analysis of OCIs before and after WAPL/CTCF co-depletion. The average pattern from the 6 and 24 h time points was used as a mask for systematic fountain identification genome-wide. (**D**) Aggregate region analysis of the 852 fountains identified using fontanka [54] in WAPL/CTCF cell line. Note that depletion of WAPL and CTCF lead to extension of the fountain pattern, evident from increased anti-diagonal interactions. (**E**) Tornado plots show the alignment of calibrated RAD21 ChIP-seq signal in WAPL/CTCF depleted cells centred at the fountain bases before and after WAPL/CTCF co-depletion for 6, 24, and 96 h.

Upon inspection of the Hi-C maps of WAPL/CTCF depleted cells, we observed that at certain sites the signal accumulates and emanates perpendicular to the diagonal in the 6 and 24 h post-depletion timepoints (Fig. 3A). Visual inspection revealed that these sites are often formed at small active chromatin domains flanked by large heterochromatic regions (Fig. 3B). We have previously named these 3D genome features ‘plumes’ [67], which are reminiscent of subsequently reported ‘flares’, ‘jets’, and ‘fountains’ [54, 68–72]. In an effort to unify the terminologies, we will refer to these features as ‘fountains’ from now on.

To systematically identify fountains, we used the fontanka tool [54], which requires a pattern mask for scanning Hi-C data. To define such a mask, we utilised our observation that fountains are formed at regions with high density of active marks flanked on either side by regions devoid of them. We thus used ATAC-seq data to identify 149 such regions, which we refer to as ‘open chromatin islands’ (OCIs, see Materials and methods). Alignment of the Hi-C signal on the OCIs showed a clear fountain pattern following depletion of WAPL/CTCF, confirming its utility for fountain calling (Fig. 3C). Genome-wide fountain annotation using fontanka with the OCI-defined Hi-C mask yielded a total of 852 fountains. Aggregate analysis of the identified fountains revealed that these regions already show a fountain-like pattern in the unperturbed condition, which is extended upon depletion of WAPL and CTCF (Fig. 3D). Notably, the number of fountains we identified far exceeds the number of chromatin jets reported in mouse CD8 thymocytes (*N* = 38) [69], and their size appears to be larger than fountains observed in zebrafish and especially nematodes (1 Mb in our data, compared to 200 and 50 kb in zebrafish and nematode, respectively) [54, 71, 72].

In addition, we found that in the unperturbed setting, fountain bases exhibit cohesin binding (Fig. 3E, Supplementary Fig. 5A). Upon removal of WAPL and CTCF, cohesin is repositioned away from the regions that make up the fountain base, as judged by the increased cohesin binding signal in the regions flanking the fountain base (Fig. 3E, Supplementary Fig. 5A). To understand the mechanism behind the cohesin dynamics around the fountain, we analysed Hi-C and RAD21 ChIP-seq data after single depletion of WAPL or CTCF [15, 26, 73]. After CTCF depletion, cohesin is still bound to the fountain base and the fountain pattern is largely unchanged (Supplementary Fig. S5B and C). This suggests that the presence of cohesin at the fountain base is not related to its retention by CTCF. On the other hand, upon WAPL depletion, cohesin is removed from the fountain base and repositioned to the flanking regions where we measured a clear enrichment in the binding signal (Supplementary Fig. S5D and E). This enrichment is likely due to CTCF-mediated extrusion blockade, and in line with this, we observed a slight fountain extension in the aggregate analysis. The reduction of cohesin at the fountain base is consistent with a reduced pool of free cohesin caused by the absence of WAPL [26], preventing re-loading of cohesin at the fountain base. Taken together, these results suggest that a combination of WAPL and CTCF activities constrains fountain formation and dynamics, and that cohesin may be directly involved in their establishment by being loaded directly at the fountain base.

### Fountains are dependent on cohesin

**Figure 4.**
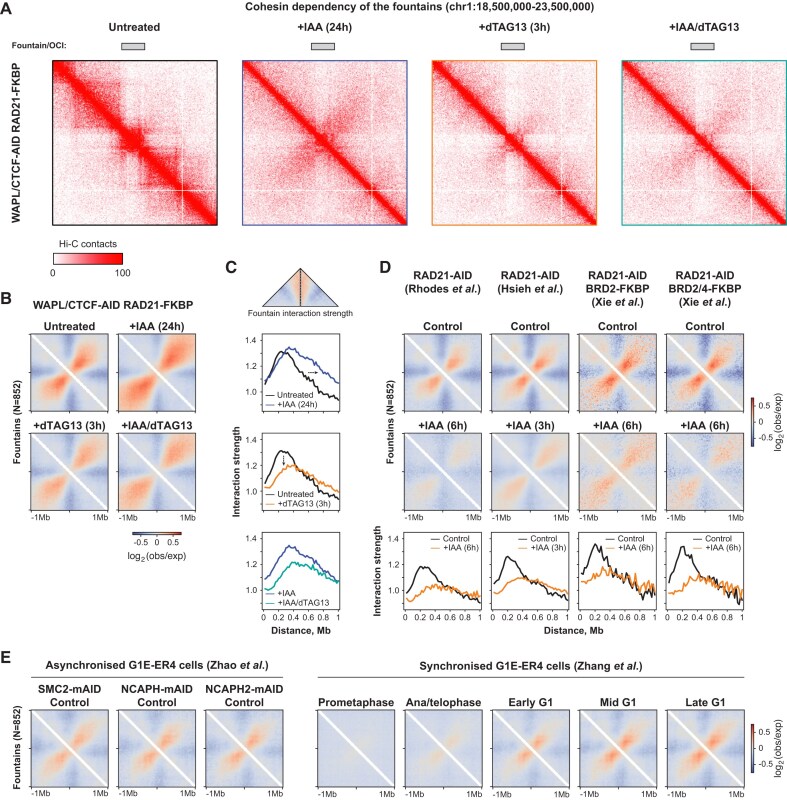
Fountains are formed by cohesin-mediated loop extrusion. (**A**) An example locus of the Hi-C contact matrices at 20 kb resolution at chromosome 1 showing the disappearance of the fountain after RAD21 depletion in the WAPL/CTCF-AID, RAD21-FKBP degron cell line following various perturbations (see Fig. 2C for schematic). (**B**) Aggregate region analysis of the fountains in WAPL/CTCF-AID, RAD21-FKBP degron cell line for different treatment conditions. Note that RAD21 depletion leads to reduced fountains both in the background of untreated and WAPL/CTCF-depleted cells. (**C**) Fountain interaction strength plots, quantifying the fountain dynamics upon WAPL/CTCF co-depletion for 24 h (top), RAD21 depletion for 3 h (middle), WAPL/CTCF co-depletion for 24 h followed by 3 h of RAD21 depletion (bottom). The arrows indicate the extension of fountain interactions upon WAPL/CTCF co-depletion and redacted fountain interactions following RAD21 depletion. (**D**) Aggregate region analysis of the fountains (top) and fountain interaction strength plots (bottom) in publicly available RAD21 degron Hi-C datasets [18, 28, 74] following 3 or 6 h of depletion, showing reduced fountains following cohesin loss. (**E**) Aggregate region analysis of the fountains in publicly available asynchronous (left) [29] and nocodazole-induced prometaphase arrest-released (right) [78] G1E-ER4 cells. Annotation of fountains is taken from WAPL/CTCF co-depleted cells. Note the fountains being present in the asynchronous cells and their gradual establishment of following the exit from mitosis.

To functionally test whether fountain formation is a consequence of cohesin-mediated loop extrusion, we used the Hi-C data from WAPL/CTCF-AID, RAD21-FKBP triple degron line (Fig. 2C), in which we can induce the formation of extended fountains upon WAPL/CTCF depletion. Following the formation of the extended fountains after 24 h of IAA treatment, we inactivated the cohesin complex for 3 h by an addition of dTAG-13. We find that loss of cohesin in untreated and following WAPL/CTCF depletion conditions results in the rapid and strong reduction of fountains (Fig. 4A). Aggregate analysis of fountains reveals that there is a genome-wide loss of both extended and regular fountains upon cohesin depletion (Fig. 4B). Quantification of fountain interaction strength shows that the majority of interactions lost upon RAD21 depletion are within 400 kb (Fig. 4C). However, co-depletion of WAPL and CTCF increases the range of interaction loss up to 1 Mb due to initial fountain extension. These results suggest that cohesin is required for fountain formation and are consistent with a model in which fountains are formed by cohesin loading and synchronised symmetric extrusion from an enhancer-rich region of the genome [54, 71, 72]. To confirm that cohesin plays a role in fountain establishment beyond our cell lines, we re-analysed published Hi-C and Micro-C data generated in mESCs following RAD21 depletion [18, 28, 74]. In four different cell lines with different depletion durations (3 or 6 h), we found that fountains are severely impaired after cohesin loss (Fig. 4D). Note that 6 h of RAD21 depletion results in a stronger decrease of fountains compared to 3 h, indicating that the complete resolution of fountains may take more than 3 h. Our analyses across different cell lines, methods and experimental settings convincingly demonstrate that cohesin plays a critical role in fountain formation.

Next, we considered the question at which point during the cell cycle fountains form. During mitosis, chromosomes are compacted and form a spiral staircase structure around a central helical scaffold [75, 76]. Subsequently, 3D genome features such as CTCF-anchored chromatin loops, TADs and compartments disappear and are then re-established following mitosis [77]. To understand the dynamics of fountain establishment, we analysed published Hi-C data for unsynchronised and nocodazole-induced prometaphase arrest-released G1E-ER4 erythroblast cells [29, 78]. While these cells represent a different cell type, we were able to partially recapitulate the fountain phenotype using fountain annotation from our mESC (Fig. 4E, left panel). We found that in prometaphase no fountains could be observed, consistent with the absence of cohesin-mediated extrusion during this phase. The first hints of fountains appear in ana/telophase, coinciding with the moment of cohesin loading after mitosis, with fountains becoming more pronounced during G1 progression (Fig. 4E, right panel). These results show that fountains are lost in metaphase but are quickly formed upon the exit from mitosis.

Finally, we investigated when fountains arise during early mouse development. To this end, we analysed two published Hi-C datasets of mouse embryogenesis [79, 80]. In zebrafish, fountains emerge after the major zygotic genome activation, which also coincides with the pluripotency stage [54, 81]. During mammalian embryogenesis, the aggregate analysis shows faint fountains as early as the two-cell stage (Supplementary Fig. S6), when zygotic genome activation takes place in the mouse [81] and 3D genome features such as TADs and compartments first appear [79, 80]. However, it is not until the pluripotent stage at E3.5 that fountains are fully established and clearly visible (Supplementary Fig. S6). These results show that also in mice, fountains are established during early development, although it is difficult to relate the time point of their formation between mammalian and non-mammalian species due to interspecific differences in the timing of ZGA and pluripotency stages.

### Fountains are found at enhancers and coincide with cohesin-regulated genes

**Figure 5.**
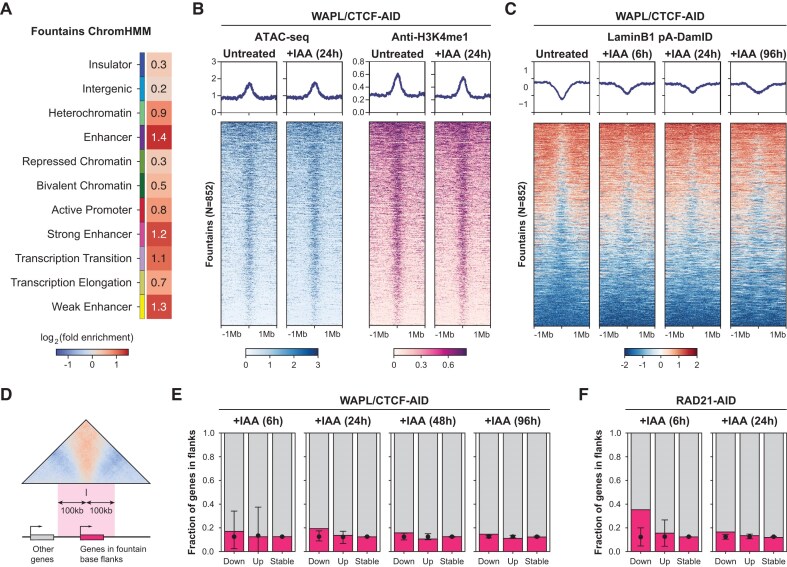
Functional characterisation of the chromatin regions associated with fountains. (**A**) ChromHMM state enrichment of the fountain bases calculated using circular permutations and chromatin states from mESCs [55]. (**B**) Tornado plots of ATAC-seq chromatin accessibility and H3K4me1 ChIP-seq centred at fountain bases before and after depletion of WAPL and CTCF for 24 h. (**C**) Tornado plots of Lamin B1 pA-DamID signal centred at fountain bases before and after depletion of WAPL and CTCF for 24 h. (**D**) Schematic for the intersection of the genes with the 100 kb fountain bases flanks. The 100 kb flanks of fountain bases were intersected with expressed genes in WAPL/CTCF-AID or RAD21-AID cell lines. Fractions of differentially expressed genes found in the fountain bases flanks (in pink) and in the rest of the genome (in grey) after WAPL/CTCF (**E**) and RAD21 (**F**) depletion. Mean fraction of genes obtained through label permutations is shown as a dot. Whiskers represent the maximum and minimum values obtained in permutations.

To understand the chromatin features that are associated with fountains, we intersected the fountain bases with the chromatin state annotation from mESCs [55]. This analysis revealed that the three most enriched features are ‘Enhancer’, ‘Strong enhancer’ and ‘Weak enhancer’ (Fig. 5A), consistent with observations in other species that fountains are formed at regions of active chromatin, specifically at enhancer regions [54, 71, 72]. To understand whether depletion of WAPL and CTCF alters the chromatin environment of the fountains, we aligned the chromatin accessibility and H3K4me1 poised enhancer histone modification data on the fountain bases. We did not observe any large-scale changes in accessibility or H3K4me1 levels upon WAPL and CTCF removal, indicating no global epigenomic differences (Fig. 5B). Next, we aligned the LaminB1 DamID data to the same regions and observed a slight increase in lamina interactions for the fountains upon WAPL/CTCF loss (Fig. 5C). Together, our observations are consistent with a model in which fountains are enhancer regions that are focally detached from nuclear periphery.

To determine whether the fountains and their expansion or loss upon WAPL/CTCF and RAD21 depletion, respectively, have any functional consequences, we performed and re-analysed time course RNA-seq experiments (Supplementary Fig. S7A) [26]. After 6 h of WAPL/CTCF or RAD21 depletion, only a limited number of genes (*N* = 57 and *N* = 130) are significantly altered in expression, while this number steadily increased to 6282 and 2822 by 96 and 24 h, respectively (FDR < 0.01, Supplementary Fig. S7B, C). Gene set enrichment analysis shows that the induced gene expression changes were consistent with previously reported transcriptional and phenotypic results following WAPL, CTCF, and RAD21 depletions (Supplementary Fig. S7D). Namely, differentiation-related and cell cycle-related categories were enriched for WAPL/CTCF and RAD21 depletions, respectively [26, 28, 60, 82].

We then intersected the different categories of genes at each time point (up-regulated, down-regulated or stable) with the position of the 100 kb fountain base flanks (Fig. 5D). We did not observe any large shift in the proportions of up- or down-regulated genes located in the fountain base flanks at any of the time points assayed after WAPL/CTCF depletion (Fig. 5E). Further separation of genes based on whether their promoters overlap with enhancer-promoter (E-P) loops or not as identified by Micro-C [28] also revealed no shifts in the proportions of genes within and outside the fountain base flanks (Supplementary Fig. S7E and F). This suggests that fountain extension upon WAPL/CTCF depletion has a mild effect on fountain-proximal genes. In contrast, the genes down-regulated following cohesin depletion were significantly enriched in the fountain base flanks (Fig. 5F), which was especially true for genes involved in the E-P loops, but not for other genes (Supplementary Fig. S7E and G). The enrichment is strongest after 6 h of cohesin depletion and largely disappears by 24 h, when secondary transcriptional effects occur, suggesting that cohesin is directly involved in the regulation of these genes. Based on these analyses, we conclude that fountains are a consequence of loop extrusion and in certain cases can drive cohesin-dependent gene activation in mESCs.

## Discussion

In this work we have used acute protein depletion techniques to better understand the dynamic changes in the 3D genome following the loss of several key 3D genome regulators. The degron mESC lines presented here enable us to refine the role that cohesin-mediated loop extrusion plays in establishing the various organisational features of chromosomes.

Loop extrusion explains why CTCF-anchored chromatin loops are formed between distally located CTCF sites and are preferentially oriented in a convergent manner [8, 13]. We found that upon loss of the cohesin release factor WAPL, CTCF-anchored chromatin loops are rapidly extended. By integrating our Hi-C with cohesin ChIP-seq data, we showed that extended loops coincide with cohesin accumulation and involve non-convergent CTCF sites in looping. Such cohesin accumulation at loop anchors may occur when an extruding complex encounters another that is already stabilised at a CTCF site, a case that is normally resolved by the continuous activity of WAPL. This is consistent with the cohesin traffic jam model proposed for loop formation in the absence of WAPL [32]. In the absence of both WAPL and CTCF, cohesin no longer accumulates at CTCF sites and all CTCF-anchored chromatin loops are simultaneously lost.

Our experiments allowed us to follow the direct and dynamic changes of compartmentalisation, which is antagonised by the loop extrusion machinery [23, 24]. Already within 6 h post-depletion we observed a severe decrease in compartmentalisation. Strikingly, when WAPL is depleted, the decrease in compartmental interactions is stronger for A compartments than for B compartments. However, when both WAPL and CTCF are depleted, this difference is no longer observed. To explain this difference, we have to consider that the vast majority of cohesin complexes are found in A compartment. Upon WAPL depletion, these complexes accumulate at CTCF binding sites, the majority of which are also located in A compartment. Therefore, cohesin complexes are largely prevented from extruding into the B compartment [83]. When CTCF is depleted together with WAPL, cohesin no longer accumulates at CTCF binding sites and extrudes into B compartments. We propose that this leads to the disruption of interactions between B compartments.

Interestingly, disruption of compartment interactions has little effect on the position of compartmental domains relative to the nuclear periphery. Given the strong anti-correlation between compartment and LaminB1 DamID scores [5], it has been hypothesised that the formation of compartments and LADs may be driven by a similar mechanism. In fact, knock-out of WAPL in HAP1 cells is associated with decreased compartmentalisation and a disruption in lamina association of LADs [24]. Here we demonstrate for the first time that in a system where both LADs and compartments are established, it is possible to perturb one without severely affecting the other. The effects observed in WAPL knockout HAP1 cells are therefore likely a consequence of the severely disrupted H3K9me3/heterochromatin domains [84], rather than the disrupted compartment interactions, which we specifically assessed in the current study using acute depletion models. Additionally, our recent study investigating the consequences of changes in cohesin positioning on nuclear lamina interactions shows that perturbation of loop extrusion regulators has little effect on global LAD positioning [60]. This provides further evidence that loop extrusion and compartmentalisation on the one hand and lamina association on the other are independent modes of genome organisation.

**Figure 6.**
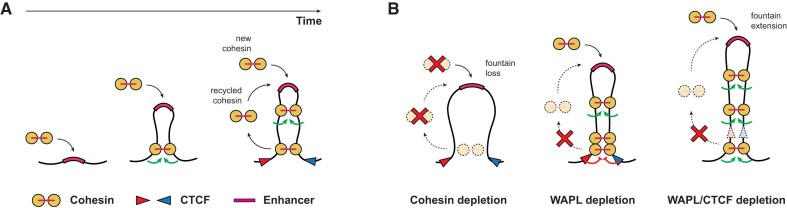
Model of the main characteristics of the fountains. (**A**) Schematic of the fountain formation process. The fountain originates at the enhancer regions (the ‘base’) by targeted loading of cohesin complexes. The fountain is gradually enlarged through loop extrusion by two different pools of cohesin: newly synthesised and recycled cohesin. The release of (extruded) cohesin results in its reloading at the base, representing a recycling mechanism. (**B**) Consequences of the depletion of the 3D genome regulators at the fountains. Cohesin depletion causes fountain dissolution (left). Depletion of WAPL leads to cohesin accumulation at CTCF sites in the flanking regions and reduced cohesin loading at the fountain base due to the lack of cohesin recycling (middle). Co-depletion of WAPL and CTCF results in unrestricted extrusion by cohesin and an increase of fountain size (right).

Using our WAPL/CTCF double degron cell line, we are able to rapidly induce fountains, a hitherto undescribed feature of chromosome organisation (Fig. 6). Fountains are located at active chromatin regions flanked by large heterochromatin domains, are strongly associated with enhancers, and exhibit cohesin binding. Our data suggest that the presence of cohesin at fountains is not due to CTCF-mediated blocking, but rather due to its loading at fountain bases, which depends on a free cohesin pool generated by WAPL. Fountains are extended after WAPL and CTCF co-depletion, which is consistent with unrestrained and continuous loop extrusion from the fountain base. Sequential degron experiments and re-analysis of published Hi-C data confirm that fountains are dependent on cohesin for their maintenance and likely also for their establishment. Active enhancers have long been hypothesised to be the preferred cohesin loading sites in the mammalian genome [85–87] and the presence of enhancers at fountain bases in mESCs further supports this hypothesis, in line with a number of recent studies [54, 69, 71, 72].

In our time course experiment, fountains were largely reduced after 96 h of WAPL and CTCF depletion. We hypothesise that this is the consequence of cohesin reloading after mitosis. During interphase, cohesin is released from chromatin by WAPL [88, 89], whereas during mitosis, cohesin is cleaved by separase via a WAPL-independent mechanism [90, 91]. In the absence of WAPL, the latter is the only mechanism of cohesin release. When WAPL and CTCF are depleted for 96 h, the length of a cell cycle is prolonged. During a longer cell cycle cohesin can extrude over longer distances, as evidenced by the substantially increased long-range genomic interactions in the RCP analysis (Supplementary Fig. S2A). Eventually, cohesin complexes loaded at fountain bases will be distributed more evenly across the genome rather than accumulating in a synchronised manner around the relatively small regions where fountains are formed. This leads to a reduction of fountains following prolonged WAPL/CTCF depletion. A prediction from this model is that fountain bases are the preferred loading sites of cohesin following mitosis, supported by our re-analysis of public data (Fig. 4D).

Intriguingly, fountain-like features have been described throughout the tree of life. In *Bacillus subtilis*, SMC complexes, which are related to cohesin, can be loaded at the origin of replication in a coordinated manner [92–94]. Following loading of SMC complexes, Hi-C reveals a cross-like organisation explained by a concerted extrusion reaction away from the replication origin [92, 93]. Similar structures, called chromatin jets, have been observed in quiescent, non-dividing mouse thymocytes [69]. Chromatin jets are formed by focal loading and diffusion of cohesin but, unlike fountains described here, can be extended by a single CTCF knockout with intact WAPL. Two recent pre-prints identified fountains in nematodes and showed that they are also cohesin-dependent, either by cleavage of the COH-1 subunit or by depletion of the SMC-3 subunit [71, 72]. In line with our findings, depletion of WAPL-1 in nematodes also leads to fountain extension [71]. Another pre-print has shown that during zygotic genome activation in early zebrafish development, fountains coincide with sites of active transcription and enhancers [54]. Interestingly, zebrafish sperm have a similar genomic structure, called flares or hinge-like domains, which are enriched for H3K27ac [68]. Finally, in *Fusarium graminearum*, jet-like domains were found upon stimulation with putrescine containing media, which triggers the activation of secondary metabolite biosynthesis genes [70]. Jet-like domains correlated with their transcriptional activity and were dependent on histone acetyltransferase activity. For the formation of zebrafish flares and *F. graminearum* jet-like domains the involvement of cohesin or other SMC motor proteins remains elusive, therefore further analysis is required to determine whether these ostensibly similar structures in different species are all formed by the same mechanism. Also, the precise mechanism of cohesin loading at fountain bases requires more detailed investigation.

What might be the function of fountains? Since fountains originate from enhancer elements, we reasoned that the extension or complete disruption of fountains should have either a positive or negative effect on gene expression. Our genome-wide transcriptome analysis in WAPL/CTCF depleted cells did not reveal a strong systematic effect on fountain-proximal gene expression, suggesting that fountain extension does not influence gene expression. Depletion of RAD21, on the other hand, showed a clear enrichment of downregulated genes around fountains, which is consistent with a model where cohesin loading at enhancers flanking fountain bases promotes enhancer communication and activates genes [25]. In *F. graminearum*, the formation of fountains or jet-like domains was also associated with the activation of proximal genes [70], although it is unclear whether the fountains are a cause or a consequence of gene activation. In *C. elegans* an opposite role for fountains is proposed, given that the loss of extrusion and fountains correlated to gene activation [72]. A recent review suggested that fountains may be a by-product of loop extrusion and have no specific function themselves, representing ‘spandrels’ of nuclear organisation [95]. In this case, fountains may be the combined consequence of cohesin loading and extrusion from enhancers and the non-random distribution of enhancers in the genome. While this is an interesting suggestion, we show here that in certain species or specific cell types fountain structures may have been exploited for gene regulation. In any case, further research is needed to gain a deeper understanding of the functional role of cohesin-mediated fountains in gene regulation and other nuclear processes.

## Supplementary Material

gkaf549_Supplemental_Files

## Data Availability

Generated ATAC-seq, ChIP-seq, RNA-seq, Hi-C, and pA-DamID data have been deposited at the GEO database (GSE181846, GSE181847, GSE181848, GSE181849, GSE181693) and are publicly available as of the date of publication. The reagents, antibodies, restriction enzymes, oligos, plasmids, and publicly available datasets used in this study are listed in Supplementary Table 1. The code for the analysis of fountains has been deposited at Zenodo (https://doi.org/10.5281/zenodo.15524791).

## References

[1] Cremer T, Cremer M Chromosome territories. Cold Spring Harb Perspect Biol. 2010; 2:a003889 10.1101/cshperspect.a003889. 20300217 PMC2829961

[2] Simonis M, Klous P, Splinter E et al. Nuclear organization of active and inactive chromatin domains uncovered by chromosome conformation capture-on-chip (4C). Nat Genet. 2006; 38:1348–54. 10.1038/ng1896. 17033623

[3] Lieberman-Aiden E, van Berkum NL, Williams L et al. Comprehensive mapping of long-range interactions reveals folding principles of the human genome. Science. 2009; 326:289–93. 10.1126/science.1181369. 19815776 PMC2858594

[4] Stevens TJ, Lando D, Basu S et al. 3D structures of individual mammalian genomes studied by single-cell hi-C. Nature. 2017; 544:59–64. 10.1038/nature21429. 28289288 PMC5385134

[5] Kind J, Pagie L, de Vries SS et al. Genome-wide maps of nuclear lamina interactions in single human cells. Cell. 2015; 163:134–47. 10.1016/j.cell.2015.08.040. 26365489 PMC4583798

[6] Dixon JR, Selvaraj S, Yue F et al. Topological domains in mammalian genomes identified by analysis of chromatin interactions. Nature. 2012; 485:376–80. 10.1038/nature11082. 22495300 PMC3356448

[7] Nora EP, Lajoie BR, Schulz EG et al. Spatial partitioning of the regulatory landscape of the X-inactivation centre. Nature. 2012; 485:381–5. 10.1038/nature11049. 22495304 PMC3555144

[8] Rao SS, Huntley MH, Durand NC et al. A 3D map of the human genome at kilobase resolution reveals principles of chromatin looping. Cell. 2014; 159:1665–80. 10.1016/j.cell.2014.11.021. 25497547 PMC5635824

[9] Davidson IF, Peters JM Genome folding through loop extrusion by SMC complexes. Nat Rev Mol Cell Biol. 2021; 22:445–64. 10.1038/s41580-021-00349-7. 33767413

[10] Wendt KS, Yoshida K, Itoh T et al. Cohesin mediates transcriptional insulation by CCCTC-binding factor. Nature. 2008; 451:796–801. 10.1038/nature06634. 18235444

[11] Parelho V, Hadjur S, Spivakov M et al. Cohesins functionally associate with CTCF on mammalian chromosome arms. Cell. 2008; 132:422–33. 10.1016/j.cell.2008.01.011. 18237772

[12] Guo Y, Xu Q, Canzio D et al. CRISPR inversion of CTCF sites alters genome topology and enhancer/promoter function. Cell. 2015; 162:900–10. 10.1016/j.cell.2015.07.038. 26276636 PMC4642453

[13] de Wit E, Vos ES, Holwerda SJ et al. CTCF binding polarity determines chromatin looping. Mol Cell. 2015; 60:676–84. 10.1016/j.molcel.2015.09.023. 26527277

[14] Sanborn AL, Rao SS, Huang SC et al. Chromatin extrusion explains key features of loop and domain formation in wild-type and engineered genomes. Proc Natl Acad Sci USA. 2015; 112:E6456–6465. 10.1073/pnas.1518552112. 26499245 PMC4664323

[15] Nora EP, Goloborodko A, Valton AL et al. Targeted degradation of CTCF decouples local insulation of chromosome domains from genomic compartmentalization. Cell. 2017; 169:930–44. 10.1016/j.cell.2017.05.004. 28525758 PMC5538188

[16] Wutz G, Varnai C, Nagasaka K et al. Topologically associating domains and chromatin loops depend on cohesin and are regulated by CTCF, WAPL, and PDS5 proteins. EMBO J. 2017; 36:3573–99. 10.15252/embj.201798004. 29217591 PMC5730888

[17] Rao SSP, Huang SC, Glenn St Hilaire B et al. Cohesin loss eliminates all loop domains. Cell. 2017; 171:305–20. 10.1016/j.cell.2017.09.026. 28985562 PMC5846482

[18] Rhodes JDP, Feldmann A, Hernandez-Rodriguez B et al. Cohesin disrupts polycomb-dependent chromosome interactions in embryonic stem cells. Cell Rep. 2020; 30:820–35. 10.1016/j.celrep.2019.12.057. 31968256 PMC6988126

[19] Fudenberg G, Imakaev M, Lu C et al. Formation of chromosomal domains by loop extrusion. Cell Rep. 2016; 15:2038–49. 10.1016/j.celrep.2016.04.085. 27210764 PMC4889513

[20] Davidson IF, Bauer B, Goetz D et al. DNA loop extrusion by human cohesin. Science. 2019; 366:1338–45. 10.1126/science.aaz3418. 31753851

[21] Kim Y, Shi Z, Zhang H et al. Human cohesin compacts DNA by loop extrusion. Science. 2019; 366:1345–9. 10.1126/science.aaz4475. 31780627 PMC7387118

[22] de Wit E, Nora EP New insights into genome folding by loop extrusion from inducible degron technologies. Nat Rev Genet. 2023; 24:73–85. 10.1038/s41576-022-00530-4. 36180596

[23] Schwarzer W, Abdennur N, Goloborodko A et al. Two independent modes of chromatin organization revealed by cohesin removal. Nature. 2017; 551:51–6. 10.1038/nature24281. 29094699 PMC5687303

[24] Haarhuis JHI, van der Weide RH, Blomen VA et al. The Cohesin release factor WAPL restricts chromatin loop extension. Cell. 2017; 169:693–707. 10.1016/j.cell.2017.04.013. 28475897 PMC5422210

[25] Hansen KL, Adachi AS, Braccioli L et al. Synergy between <em>cis</em>-regulatory elements can render cohesin dispensable for distal enhancer function. bioRxiv4 October 2024, preprint: not peer reviewed 10.1101/2024.10.04.615095. PMC13446962 41308125

[26] Liu NQ, Maresca M, van den Brand T et al. WAPL maintains a cohesin loading cycle to preserve cell-type-specific distal gene regulation. Nat Genet. 2021; 53:100–9. 10.1038/s41588-020-00744-4. 33318687 PMC7610352

[27] Magnitov M, de Wit E Attraction and disruption: how loop extrusion and compartmentalisation shape the nuclear genome. Curr Opin Genet Dev. 2024; 86:102194 10.1016/j.gde.2024.102194. 38636335 PMC11190842

[28] Hsieh TS, Cattoglio C, Slobodyanyuk E et al. Enhancer-promoter interactions and transcription are largely maintained upon acute loss of CTCF, cohesin, WAPL or YY1. Nat Genet. 2022; 54:1919–32. 10.1038/s41588-022-01223-8. 36471071 PMC9729117

[29] Zhao H, Lin Y, Lin E et al. Genome folding principles uncovered in condensin-depleted mitotic chromosomes. Nat Genet. 2024; 56:1213–24.10.1038/s41588-024-01759-x.38802567

[30] Lam JC, Aboreden NG, Midla SC et al. YY1-controlled regulatory connectivity and transcription are influenced by the cell cycle. Nat Genet. 2024; 56:1938–52. 10.1038/s41588-024-01871-y. 39210046 PMC11687402

[31] Aboreden NG, Zhao H, Shan F et al. Cis-regulatory chromatin contacts form de novo in the absence of loop extrusion. bioRxiv13 January 2025, preprint: not peer reviewed 10.1101/2025.01.12.632634.

[32] Allahyar A, Vermeulen C, Bouwman BAM et al. Enhancer hubs and loop collisions identified from single-allele topologies. Nat Genet. 2018; 50:1151–60. 10.1038/s41588-018-0161-5. 29988121

[33] Liu NQ, Ter Huurne M, Nguyen LN et al. The non-coding variant rs1800734 enhances DCLK3 expression through long-range interaction and promotes colorectal cancer progression. Nat Commun. 2017; 8:14418 10.1038/ncomms14418. 28195176 PMC5316867

[34] van Schaik T, Vos M, Peric-Hupkes D et al. Cell cycle dynamics of lamina-associated DNA. EMBO Rep. 2020; 21:e50636 10.15252/embr.202050636. 32893442 PMC7645246

[35] Langmead B, Salzberg SL Fast gapped-read alignment with Bowtie 2. Nat Methods. 2012; 9:357–9. 10.1038/nmeth.1923. 22388286 PMC3322381

[36] Li H, Durbin R Fast and accurate short read alignment with Burrows-Wheeler transform. Bioinformatics. 2009; 25:1754–60. 10.1093/bioinformatics/btp324. 19451168 PMC2705234

[37] Ramirez F, Ryan DP, Gruning B et al. deepTools2: a next generation web server for deep-sequencing data analysis. Nucleic Acids Res. 2016; 44:W160–5. 10.1093/nar/gkw257. 27079975 PMC4987876

[38] Zhang Y, Liu T, Meyer CA et al. Model-based analysis of ChIP-Seq (MACS). Genome Biol. 2008; 9:R137 10.1186/gb-2008-9-9-r137. 18798982 PMC2592715

[39] Grant CE, Bailey TL, Noble WS FIMO: scanning for occurrences of a given motif. Bioinformatics. 2011; 27:1017–8. 10.1093/bioinformatics/btr064. 21330290 PMC3065696

[40] Fornes O, Castro-Mondragon JA, Khan A et al. JASPAR 2020: update of the open-access database of transcription factor binding profiles. Nucleic Acids Res. 2020; 48:D87–92. 31701148 10.1093/nar/gkz1001 PMC7145627

[41] Dobin A, Davis CA, Schlesinger F et al. STAR: ultrafast universal RNA-seq aligner. Bioinformatics. 2013; 29:15–21. 10.1093/bioinformatics/bts635. 23104886 PMC3530905

[42] Frankish A, Diekhans M, Jungreis I et al. Gencode 2021. Nucleic Acids Res. 2021; 49:D916–23. 10.1093/nar/gkaa1087. 33270111 PMC7778937

[43] Love MI, Huber W, Anders S Moderated estimation of fold change and dispersion for RNA-seq data with DESeq2. Genome Biol. 2014; 15:550 10.1186/s13059-014-0550-8. 25516281 PMC4302049

[44] Subramanian A, Tamayo P, Mootha VK et al. Gene set enrichment analysis: a knowledge-based approach for interpreting genome-wide expression profiles. Proc Natl Acad Sci USA. 2005; 102:15545–50. 10.1073/pnas.0506580102. 16199517 PMC1239896

[45] Castanza AS, Recla JM, Eby D et al. Extending support for mouse data in the Molecular Signatures Database (MSigDB). Nat Methods. 2023; 20:1619–20. 10.1038/s41592-023-02014-7. 37704782 PMC11397807

[46] Open2C Abdennur N, Fudenberg G, Flyamer IM et al. Bioframe: operations on genomic intervals in Pandas dataframes. Bioinformatics. 2024; 40:btae088 10.1093/bioinformatics/btae088. 38402507 PMC10903647

[47] Servant N, Varoquaux N, Lajoie BR et al. HiC-Pro: an optimized and flexible pipeline for Hi-C data processing. Genome Biol. 2015; 16:259 10.1186/s13059-015-0831-x. 26619908 PMC4665391

[48] Open2C Abdennur N, Fudenberg G, Flyamer IM et al. Pairtools: from sequencing data to chromosome contacts. PLoS Comput Biol. 2024; 20:e1012164. 38809952 10.1371/journal.pcbi.1012164 PMC11164360

[49] Abdennur N, Mirny LA Cooler: scalable storage for Hi-C data and other genomically labeled arrays. Bioinformatics. 2020; 36:311–6. 10.1093/bioinformatics/btz540. 31290943 PMC8205516

[50] Open2C Abdennur N, Abraham S, Fudenberg G et al. Cooltools: enabling high-resolution hi-C analysis in Python. PLoS Comput Biol. 2024; 20:e1012067. 38709825 10.1371/journal.pcbi.1012067 PMC11098495

[51] Bonev B, Mendelson Cohen N, Szabo Q et al. Multiscale 3D genome rewiring during mouse neural development. Cell. 2017; 171:557–72. 10.1016/j.cell.2017.09.043. 29053968 PMC5651218

[52] Flyamer IM, Illingworth RS, Bickmore WA Coolpup.Py: versatile pile-up analysis of hi-C data. Bioinformatics. 2020; 36:2980–5. 10.1093/bioinformatics/btaa073. 32003791 PMC7214034

[53] van der Weide RH, van den Brand T, Haarhuis JHI et al. Hi-C analyses with GENOVA: a case study with cohesin variants. NAR Genom Bioinform. 2021; 3:lqab040 10.1093/nargab/lqab040. 34046591 PMC8140737

[54] Galitsyna A, Ulianov SV, Bykov NS et al. Extrusion fountains are hallmarks of chromosome organization emerging upon zygotic genome activation. bioRxiv15 July 2023, preprint: not peer reviewed 10.1101/2023.07.15.549120. PMC13018191 41690937

[55] Pintacuda G, Wei G, Roustan C et al. hnRNPK recruits PCGF3/5-PRC1 to the xist RNA B-repeat to establish polycomb-mediated chromosomal silencing. Mol Cell. 2017; 68:955–69. 10.1016/j.molcel.2017.11.013. 29220657 PMC5735038

[56] Gel B, Diez-Villanueva A, Serra E et al. regioneR: an R/bioconductor package for the association analysis of genomic regions based on permutation tests. Bioinformatics. 2016; 32:289–91. 10.1093/bioinformatics/btv562. 26424858 PMC4708104

[57] Martin M Cutadapt removes adapter sequences from high-throughput sequencing reads. EMBnet j. 2011; 17:10–2. 10.14806/ej.17.1.200.

[58] Nishimura K, Fukagawa T, Takisawa H et al. An auxin-based degron system for the rapid depletion of proteins in nonplant cells. Nat Methods. 2009; 6:917–22. 10.1038/nmeth.1401. 19915560

[59] Busslinger GA, Stocsits RR, van der Lelij P et al. Cohesin is positioned in mammalian genomes by transcription, CTCF and wapl. Nature. 2017; 544:503–7. 10.1038/nature22063. 28424523 PMC6080695

[60] van Schaik T, Liu NQ, Manzo SG et al. CTCF and cohesin promote focal detachment of DNA from the nuclear lamina. Genome Biol. 2022; 23:185 10.1186/s13059-022-02754-3. 36050765 PMC9438259

[61] Gassler J, Brandao HB, Imakaev M et al. A mechanism of cohesin-dependent loop extrusion organizes zygotic genome architecture. EMBO J. 2017; 36:3600–18. 10.15252/embj.201798083. 29217590 PMC5730859

[62] Anania C, Acemel RD, Jedamzick J et al. In vivo dissection of a clustered-CTCF domain boundary reveals developmental principles of regulatory insulation. Nat Genet. 2022; 54:1026–36. 10.1038/s41588-022-01117-9. 35817979 PMC9279147

[63] Han R, Huang Y, Robers M et al. Detailed <em>in vivo</em>characterization of the players and impact of individual loop extrusion trajectories. bioRxiv8 November 2024, preprint: not peer reviewed 10.1101/2023.01.04.522689.

[64] Imakaev M, Fudenberg G, McCord RP et al. Iterative correction of hi-C data reveals hallmarks of chromosome organization. Nat Methods. 2012; 9:999–1003. 10.1038/nmeth.2148. 22941365 PMC3816492

[65] Nabet B, Roberts JM, Buckley DL et al. The dTAG system for immediate and target-specific protein degradation. Nat Chem Biol. 2018; 14:431–41. 10.1038/s41589-018-0021-8. 29581585 PMC6295913

[66] Maresca M, Liu NQ, de Wit E Acute protein depletion strategies to functionally dissect the 3D genome. Methods Mol Biol. 2022; 2532:311–31. 35867256 10.1007/978-1-0716-2497-5_15

[67] Liu NQ, Magnitov M, Schijns M et al. Rapid depletion of CTCF and cohesin proteins reveals dynamic features of chromosome architecture. bioRxiv27 August 2021, preprint: not peer reviewed 10.1101/2021.08.27.457977.

[68] Wike CL, Guo Y, Tan M et al. Chromatin architecture transitions from zebrafish sperm through early embryogenesis. Genome Res. 2021; 31:981–94. 10.1101/gr.269860.120. 34006569 PMC8168589

[69] Guo Y, Al-Jibury E, Garcia-Millan R et al. Chromatin jets define the properties of cohesin-driven in vivo loop extrusion. Mol Cell. 2022; 82:3769–80. 10.1016/j.molcel.2022.09.003. 36182691

[70] Shao W, Wang J, Zhang Y et al. The jet-like chromatin structure defines active secondary metabolism in fungi. Nucleic Acids Res. 2024; 52:4906–21. 10.1093/nar/gkae131. 38407438 PMC11109943

[71] Kim J, Wang H, Ercan S Cohesin organizes 3D DNA contacts surrounding active enhancers in C. elegans. Genome Res. 2025; 35:1108–23. 10.1101/gr.279365.124. 40210441 PMC12047539

[72] Isiaka BN, Semple JI, Haemmerli A et al. Cohesin forms fountains at active enhancers in <em>C. elegans</em>. bioRxiv16 July 2023, preprint: not peer reviewed 10.1101/2023.07.14.549011.

[73] Nora EP, Caccianini L, Fudenberg G et al. Molecular basis of CTCF binding polarity in genome folding. Nat Commun. 2020; 11:5612 10.1038/s41467-020-19283-x. 33154377 PMC7645679

[74] Xie L, Dong P, Qi Y et al. BRD2 compartmentalizes the accessible genome. Nat Genet. 2022; 54:481–91. 10.1038/s41588-022-01044-9. 35410381 PMC9099420

[75] Naumova N, Imakaev M, Fudenberg G et al. Organization of the mitotic chromosome. Science. 2013; 342:948–53. 10.1126/science.1236083. 24200812 PMC4040465

[76] Gibcus JH, Samejima K, Goloborodko A et al. A pathway for mitotic chromosome formation. Science. 2018; 359:eaao6135 10.1126/science.aao6135. 29348367 PMC5924687

[77] Nagano T, Lubling Y, Varnai C et al. Cell-cycle dynamics of chromosomal organization at single-cell resolution. Nature. 2017; 547:61–7. 10.1038/nature23001. 28682332 PMC5567812

[78] Zhang H, Emerson DJ, Gilgenast TG et al. Chromatin structure dynamics during the mitosis-to-G1 phase transition. Nature. 2019; 576:158–62. 10.1038/s41586-019-1778-y. 31776509 PMC6895436

[79] Du Z, Zheng H, Huang B et al. Allelic reprogramming of 3D chromatin architecture during early mammalian development. Nature. 2017; 547:232–5. 10.1038/nature23263. 28703188

[80] Ke Y, Xu Y, Chen X et al. 3D Chromatin structures of mature gametes and structural reprogramming during mammalian embryogenesis. Cell. 2017; 170:367–81. 10.1016/j.cell.2017.06.029. 28709003

[81] Paranjpe SS, Veenstra GJ Establishing pluripotency in early development. Biochim Biophys Acta. 2015; 1849:626–36. 10.1016/j.bbagrm.2015.03.006. 25857441 PMC4437833

[82] Kubo N, Ishii H, Xiong X et al. Promoter-proximal CTCF binding promotes distal enhancer-dependent gene activation. Nat Struct Mol Biol. 2021; 28:152–61. 10.1038/s41594-020-00539-5. 33398174 PMC7913465

[83] Spracklin G, Abdennur N, Imakaev M et al. Diverse silent chromatin states modulate genome compartmentalization and loop extrusion barriers. Nat Struct Mol Biol. 2023; 30:38–51. 10.1038/s41594-022-00892-7. 36550219 PMC9851908

[84] Haarhuis JHI, van der Weide RH, Blomen VA et al. A mediator-cohesin axis controls heterochromatin domain formation. Nat Commun. 2022; 13:754 10.1038/s41467-022-28377-7. 35136067 PMC8826356

[85] Valton AL, Venev SV, Mair B et al. A cohesin traffic pattern genetically linked to gene regulation. Nat Struct Mol Biol. 2022; 29:1239–51. 10.1038/s41594-022-00890-9. 36482254 PMC10228515

[86] Vos ESM, Valdes-Quezada C, Huang Y et al. Interplay between CTCF boundaries and a super enhancer controls cohesin extrusion trajectories and gene expression. Mol Cell. 2021; 81:3082–95. 10.1016/j.molcel.2021.06.008. 34197738

[87] Rinzema NJ, Sofiadis K, Tjalsma SJD et al. Building regulatory landscapes reveals that an enhancer can recruit cohesin to create contact domains, engage CTCF sites and activate distant genes. Nat Struct Mol Biol. 2022; 29:563–74. 10.1038/s41594-022-00787-7. 35710842 PMC9205769

[88] Shintomi K, Hirano T Releasing cohesin from chromosome arms in early mitosis: opposing actions of Wapl-Pds5 and Sgo1. Genes Dev. 2009; 23:2224–36. 10.1101/gad.1844309. 19696148 PMC2751989

[89] Tedeschi A, Wutz G, Huet S et al. Wapl is an essential regulator of chromatin structure and chromosome segregation. Nature. 2013; 501:564–8. 10.1038/nature12471. 23975099 PMC6080692

[90] Uhlmann F, Lottspeich F, Nasmyth K Sister-chromatid separation at anaphase onset is promoted by cleavage of the cohesin subunit Scc1. Nature. 1999; 400:37–42. 10.1038/21831. 10403247

[91] Hauf S, Waizenegger IC, Peters JM Cohesin cleavage by separase required for anaphase and cytokinesis in human cells. Science. 2001; 293:1320–3. 10.1126/science.1061376. 11509732

[92] Wang X, Brandao HB, Le TB et al. Bacillus subtilis SMC complexes juxtapose chromosome arms as they travel from origin to terminus. Science. 2017; 355:524–7. 10.1126/science.aai8982. 28154080 PMC5484144

[93] Wang X, Hughes AC, Brandao HB et al. *In v**ivo* evidence for ATPase-dependent DNA translocation by the Bacillus subtilis SMC condensin complex. Mol Cell. 2018; 71:841–7. 10.1016/j.molcel.2018.07.006. 30100265 PMC6591583

[94] Banigan EJ, van den Berg AA, Brandao HB et al. Chromosome organization by one-sided and two-sided loop extrusion. eLife. 2020; 9:e53558 10.7554/eLife.53558. 32250245 PMC7295573

[95] Solovei I, Mirny L Spandrels of the cell nucleus. Curr Opin Cell Biol. 2024; 90:102421 10.1016/j.ceb.2024.102421. 39180905

