# Supplementary material for: Extrusion fountains are restricted by WAPL-dependent cohesin release and CTCF barriers
Source: Nucleic Acids Res. 2025 Jun 30;53(12):gkaf549. doi: 10.1093/nar/gkaf549 (PMC12207409; doi:10.1093/nar/gkaf549)
Supplement: gkaf549_Supplemental_Files [file gkaf549_supplemental_files.zip › Supplementary_Information.pdf]

Supplementary Information

Extrusion fountains are restricted by WAPL-dependent cohesin release and CTCF barriers

Ning Qing Liu†, Mikhail Magnitov†, Marijne Schijns, Tom van Schaik, Hans Teunissen, Bas van Steensel, Elzo de Wit

Figure S1

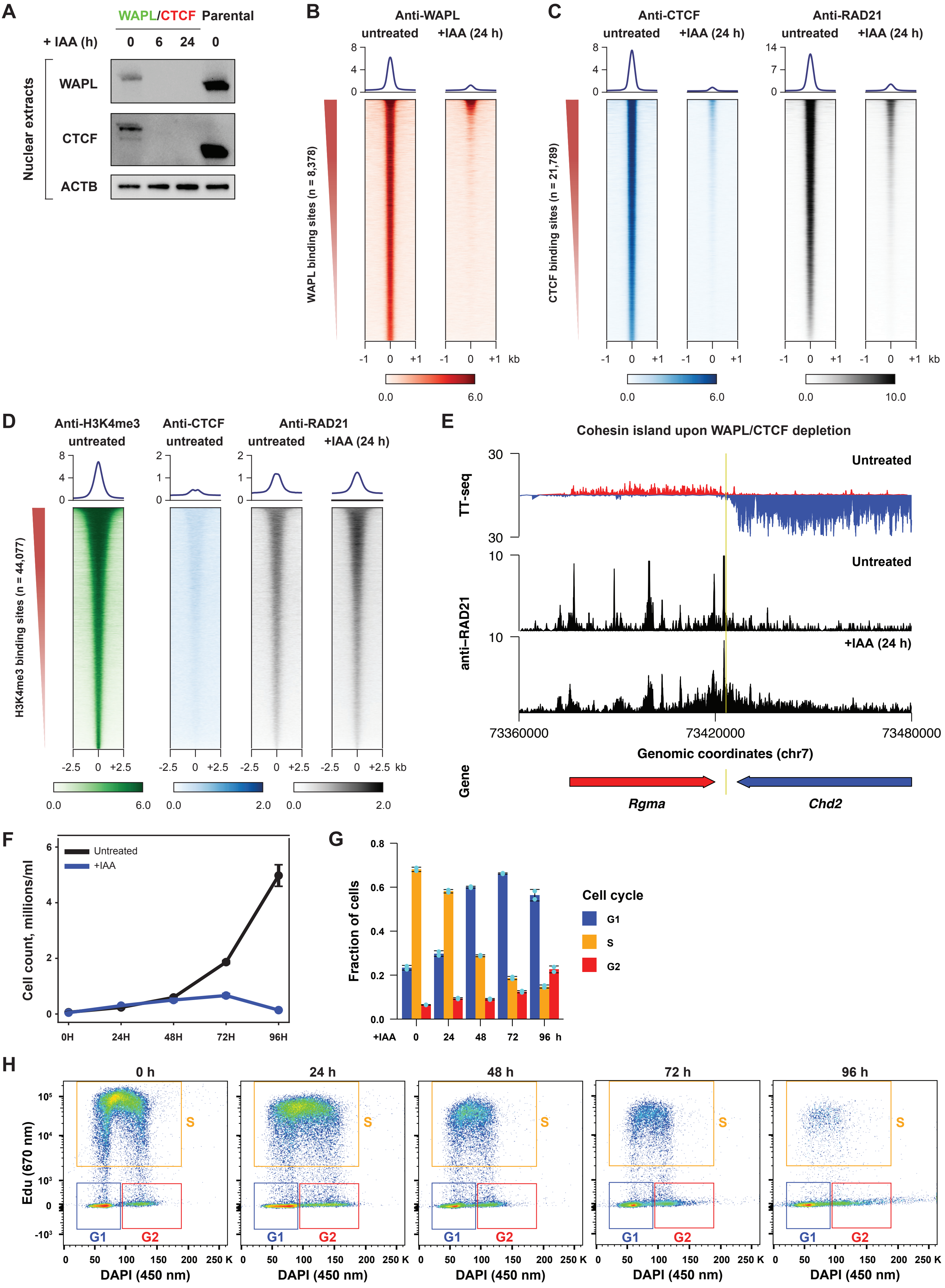

A
WAPL/CTCF
Parental
+ IAA (h)
0
6
24
0
Nuclear extracts
WAPL
CTCF
ACTB
B
Anti-WAPL
untreated
+IAA (24 h)
WAPL binding sites (n = 8,378)
-1
0
+1
kb
0.0
6.0
C
Anti-CTCF
Anti-RAD21
untreated
+IAA (24 h)
CTCF binding sites (n = 21,789)
0.0
6.0
0.0
10.0
D
Anti-H3K4me3
Anti-CTCF
Anti-RAD21
untreated
+IAA (24 h)
H3K4me3 binding sites (n = 44,077)
-2.5
0
+2.5
kb
0.0
6.0
0.0
2.0
0.0
2.0
E
Cohesin island upon WAPL/CTCF depletion
TT-seq
Untreated
anti-RAD21
Untreated
+IAA (24 h)
73360000
73420000
73480000
Genomic coordinates (chr7)
Gene
Rgma
Chd2
F
Untreated
+IAA
Cell count, millions/ml
0H
24H
48H
72H
96H
G
Fraction of cells
+IAA
0
24
48
72
96 h
Cell cycle
G1
S
G2
H
0 h
24 h
48 h
72 h
96 h
Edu (670 nm)
DAPI (450 nm)
S
G1
G2

Figure S1. Molecular characterisation of the WAPL/CTCF-AID cell line. (A) Western blot analysis of WAPL and CTCF upon IAA treatment. ChIP-seq analysis of WAPL **(B)**, CTCF and RAD21 **(C)** binding after IAA treatment. **(D)** Tornado plots showing the aligned RAD21 ChIP-seq signal at active promoters marked by strong H3K4me3 levels with and without WAPL and CTCF. **(E)** Cohesin accumulation at a "cohesin island" (1) in between two convergently transcribed genes. **(F)** Growth curve showing the total cell number of untreated and IAA-treated cells at different time points. Dots indicate mean values. Error bars indicate standard deviation. **(G)** Quantification of cycle phases based on DAPI/EdU flow cytometry analysis for various times after WAPL/CTCF depletion by IAA treatment. Data are from two technical replicate experiments. Mean values (bars), standard deviation (error bars) and individual replicates are visualized. All single cells are gated, and G1, S and G2 cells are counted. **(H)** Representative flow cytometry data plots showing EdU incorporation to measure the cycle phases of individual cells.

Figure S2

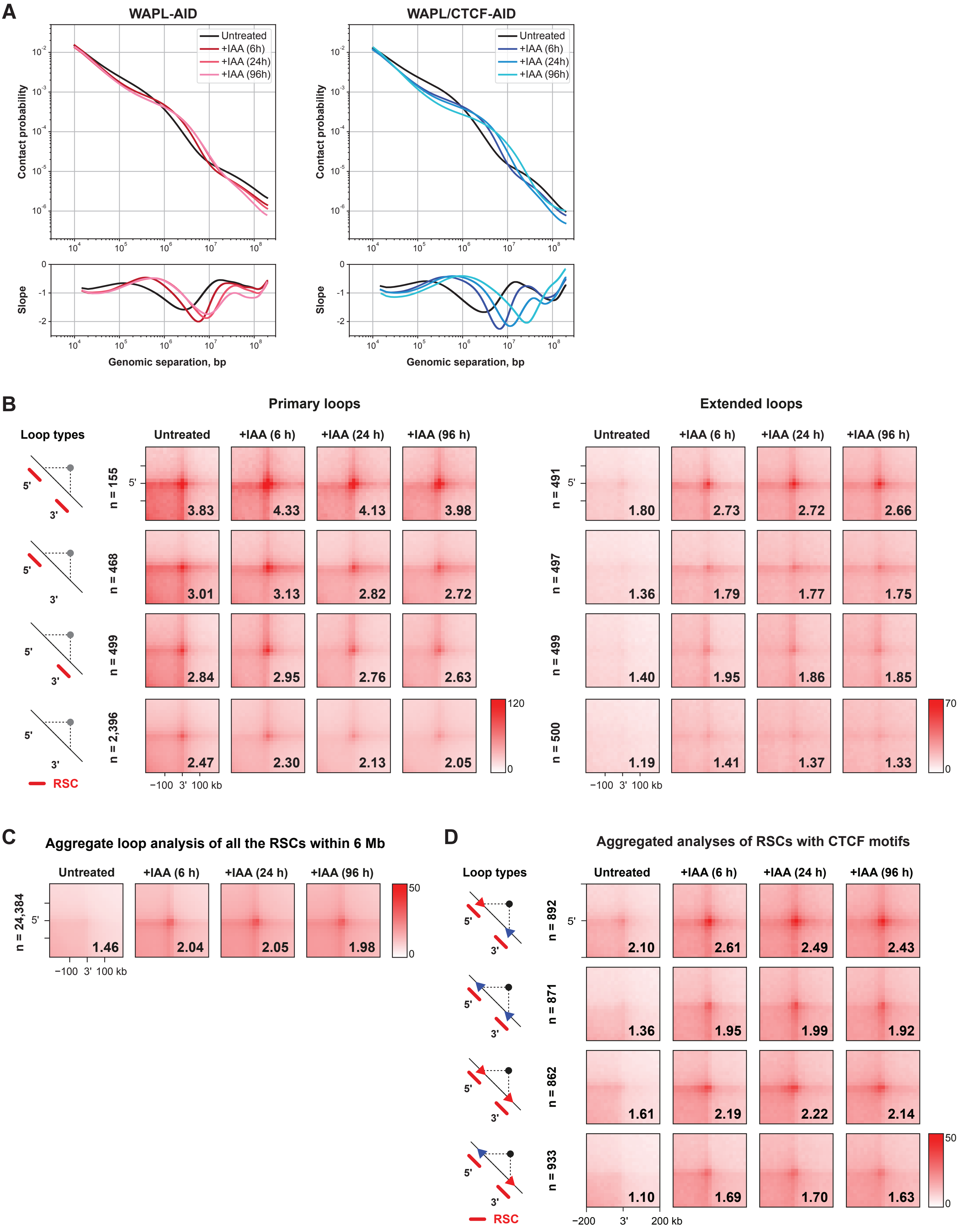
A
WAPL-AID
WAPL/CTCF-AID
Untreated
+IAA (6h)
+IAA (24h)
+IAA (96h)
Contact probability
Slope
Genomic separation, bp
B
Primary loops
Extended loops
Loop types
Untreated
+IAA (6 h)
+IAA (24 h)
+IAA (96 h)
n = 155
3.83
4.33
4.13
3.98
n = 491
1.80
2.73
2.72
2.66
n = 468
3.01
3.13
2.82
2.72
n = 497
1.36
1.79
1.77
1.75
n = 499
2.84
2.95
2.76
2.63
n = 499
1.40
1.95
1.86
1.85
n = 2,396
2.47
2.30
2.13
2.05
n = 500
1.19
1.41
1.37
1.33
120
0
70
0
RSC
−100 3' 100 kb
C
Aggregate loop analysis of all the RSCs within 6 Mb
n = 24,384
1.46
2.04
2.05
1.98
50
0
−100 3' 100 kb
D
Aggregated analyses of RSCs with CTCF motifs
n = 892
2.10
2.61
2.49
2.43
n = 871
1.36
1.95
1.99
1.92
n = 862
1.61
2.19
2.22
2.14
n = 933
1.10
1.69
1.70
1.63
50
0
RSC
−200 3' 200 kb

Figure S2. Analyses of features of chromosome organisation after WAPL and WAPL/CTCF depletion. (A) Relative contact probability (top panel) and its derivative (bottom panel) calculated from the Hi-C matrices of WAPL and WAPL/CTCF depleted cells. **(B)** Quantification of the primary and extended loops in the WAPL depletion using aggregate peak analysis. Loops are stratified by the presence of an RSC at the loop anchor. **(C)** Quantification of putative *in silico* generated chromatin loops formed by two RSCs within 6Mb. **(D)** Quantification of the chromatin loops between two RSCs containing CTCF sites with unique orientations in the WAPL depletion. The distance of two RSCs is restricted up to 6Mb. Values in the bottom-right corner indicate the interaction strength of the loops and TADs over the background.

Figure S3

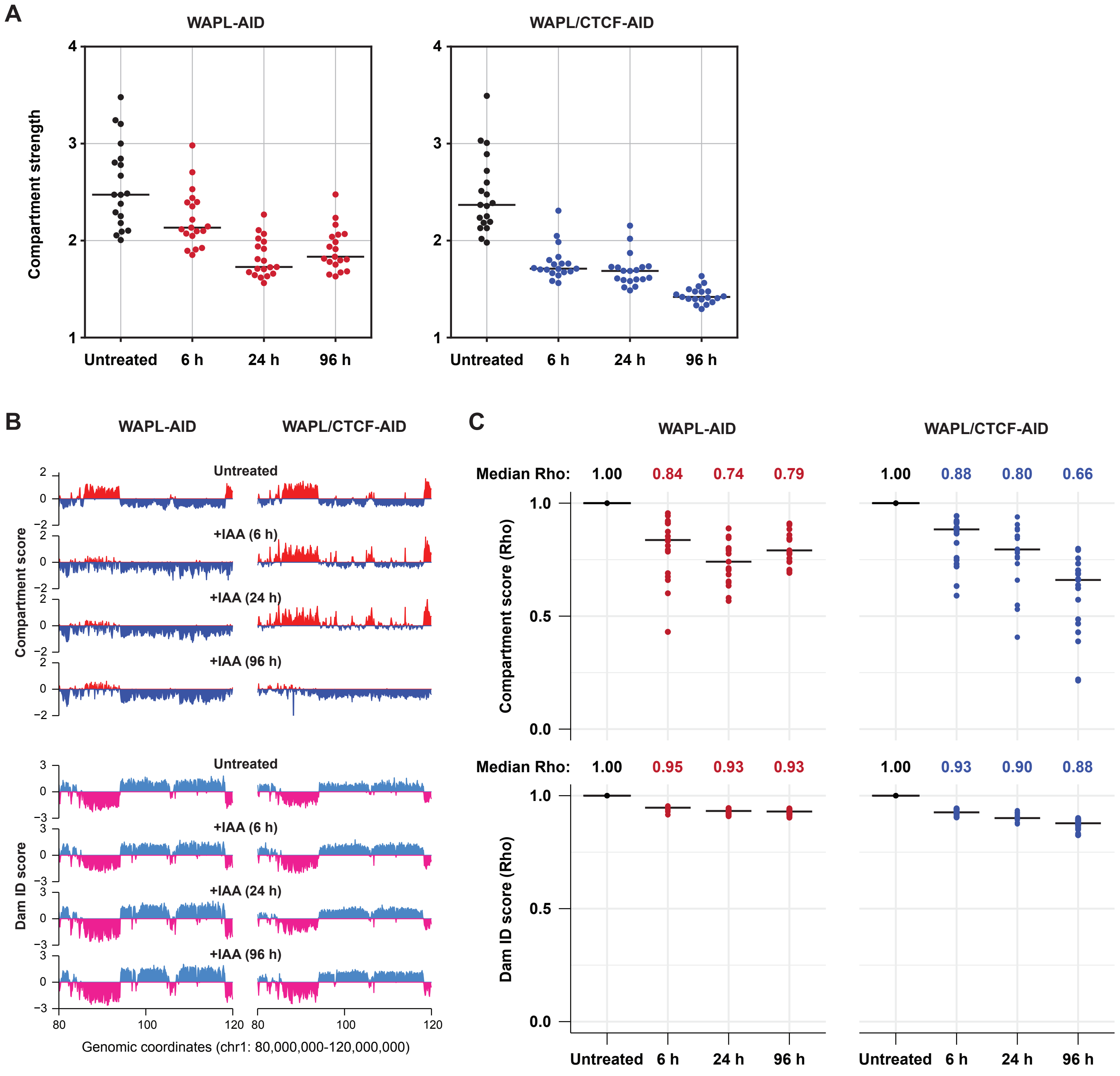
A
WAPL-AID
WAPL/CTCF-AID
Compartment strength
Untreated
6 h
24 h
96 h
B
WAPL-AID
WAPL/CTCF-AID
Compartment score
Untreated
+IAA (6 h)
+IAA (24 h)
+IAA (96 h)
Dam ID score
Genomic coordinates (chr1: 80,000,000-120,000,000)
C
WAPL-AID
WAPL/CTCF-AID
Median Rho: 1.00 0.84 0.74 0.79
1.00 0.88 0.80 0.66
Compartment score (Rho)
Median Rho: 1.00 0.95 0.93 0.93
1.00 0.93 0.90 0.88
Dam ID score (Rho)

Figure S3. Compartmentalisation changes are uncoupled from nuclear lamina interactions. (A) Genome-wide quantification of compartment strength following WAPL and WAPL/CTCF depletion. Each point represents a value for one chromosome. Black lines indicate the mean values. **(B)** An example region showing that changes in compartmentalisation (represented by compartment scores identified for Hi-C matrices) has only subtle effects on nuclear lamina association (represented by Lamin B1 pA-DamID scores). **(C)** Spearman correlation coefficients between the compartment scores (top) and Lamin B1 pA-DamID scores (bottom) in the untreated cells versus all the time course points. Each point represents a value for one chromosome. Black lines indicate the mean values.

Figure S4

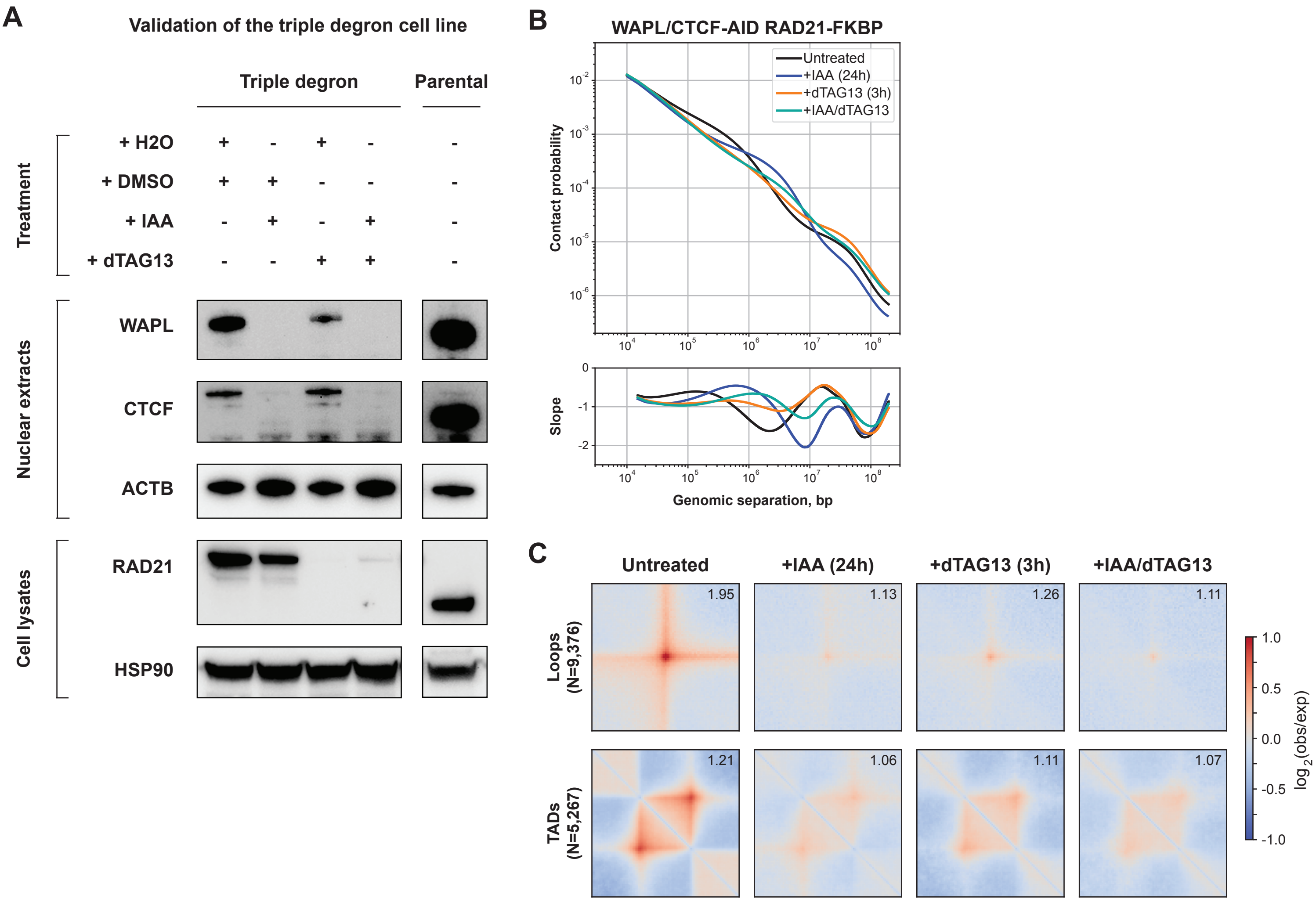
A
Validation of the triple degron cell line
Triple degron
Parental
Treatment
+ H2O
+ DMSO
+ IAA
+ dTAG13
Nuclear extracts
WAPL
CTCF
ACTB
Cell lysates
RAD21
HSP90
B
WAPL/CTCF-AID RAD21-FKBP
Untreated
+IAA (24h)
+dTAG13 (3h)
+IAA/dTAG13
Contact probability
Slope
Genomic separation, bp
C
Untreated
+IAA (24h)
+dTAG13 (3h)
+IAA/dTAG13
Loops (N=9,376)
TADs (N=5,267)
1.95
1.13
1.26
1.11
1.21
1.06
1.11
1.07
log2(obs/exp)
1.0
0.5
0.0
-0.5
-1.0

Figure S4. Molecular characterisation of the WAPL/CTCF-AID, RAD21-FKBP cell line. **(A)** Western blot analysis of WAPL, CTCF, and RAD21 after IAA and dTAG13 treatments in the triple degron cell line. **(B)** Relative contact probability (top panel) and its derivative (bottom panel) calculated from the Hi-C matrices following different treatments in the triple degron cell line. **(C)** Aggregate loop and TAD analysis for treatment conditions in the triple degron cell line. Values in the upper-right corner indicate the interaction strength of the loops and TADs over the background. The loops and TADs are obtained from (2).

Figure S5

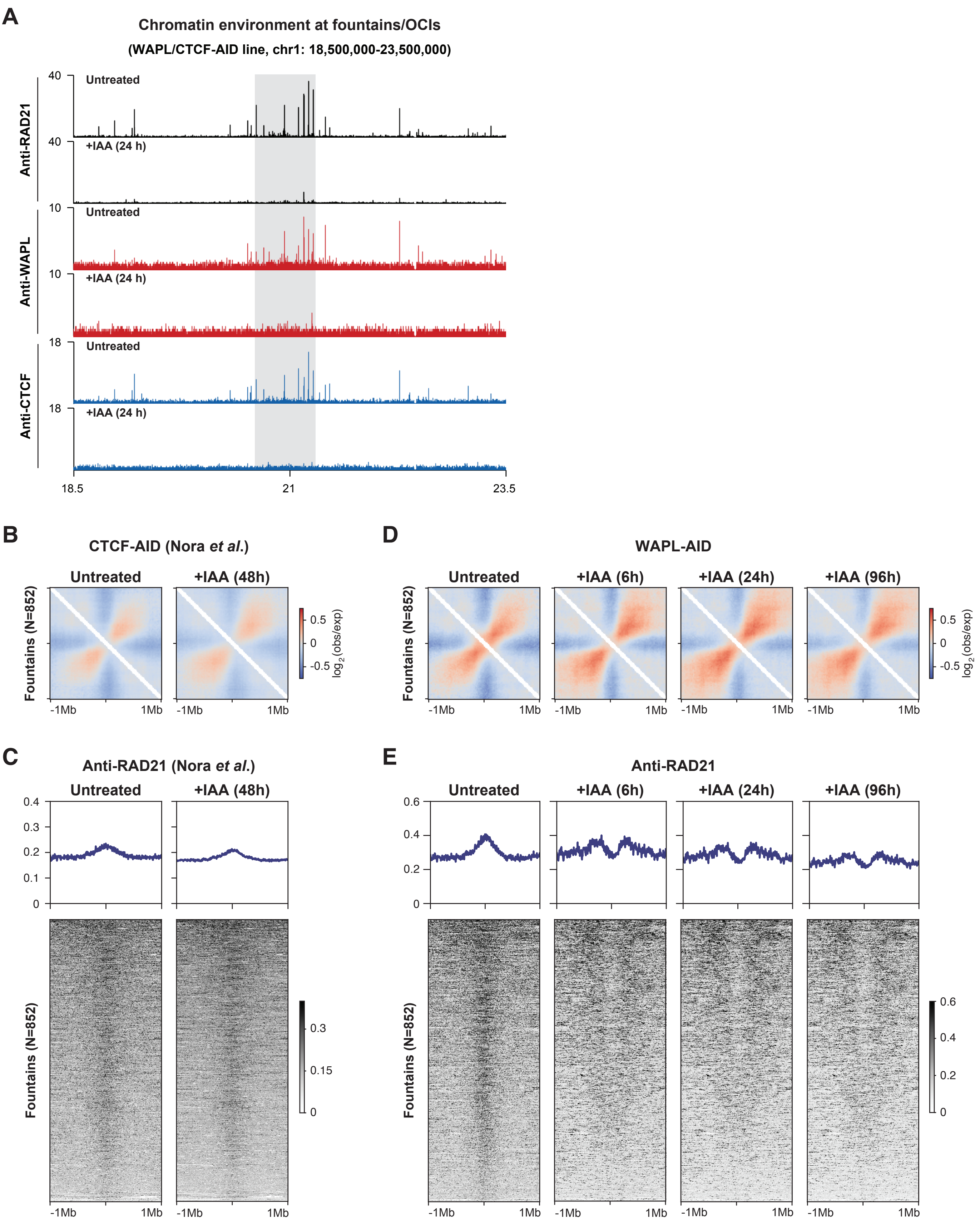
A
Chromatin environment at fountains/OCIs
(WAPL/CTCF-AID line, chr1: 18,500,000-23,500,000)
Anti-RAD21
Anti-WAPL
Anti-CTCF
Untreated
+IAA (24 h)
40
10
18
18.5
21
23.5
B
CTCF-AID (Nora et al.)
Untreated
+IAA (48h)
Fountains (N=852)
-1Mb
1Mb
0.5
0
-0.5
log2(obs/exp)
C
Anti-RAD21 (Nora et al.)
0.4
0.3
0.2
0.1
0.15
D
WAPL-AID
+IAA (6h)
+IAA (24h)
+IAA (96h)
E
Anti-RAD21
0.6

Figure S5. WAPL and CTCF constrain cohesin at the fountains. (A) Chromatin environment of a fountain showing RAD21, WAPL and CTCF ChIP-seq signals in WAPL/CTCF-AID cell line before and after WAPL/CTCF co-depletion. Grey rectangle indicates the position of the fountain. Aggregate region analysis of fountains and tornado plots of calibrated RAD21 ChIP-seq signal in CTCF **(B, C)** or WAPL **(D, E)** single depletions. Note that after CTCF depletion cohesin signal around fountain is unaffected, while after WAPL depletion cohesin is repositioned to the flanking regions. Annotation of fountains is taken from WAPL/CTCF co-depleted cells. The data for the CTCF-AID cell line is from (3,4).

Figure S6

A

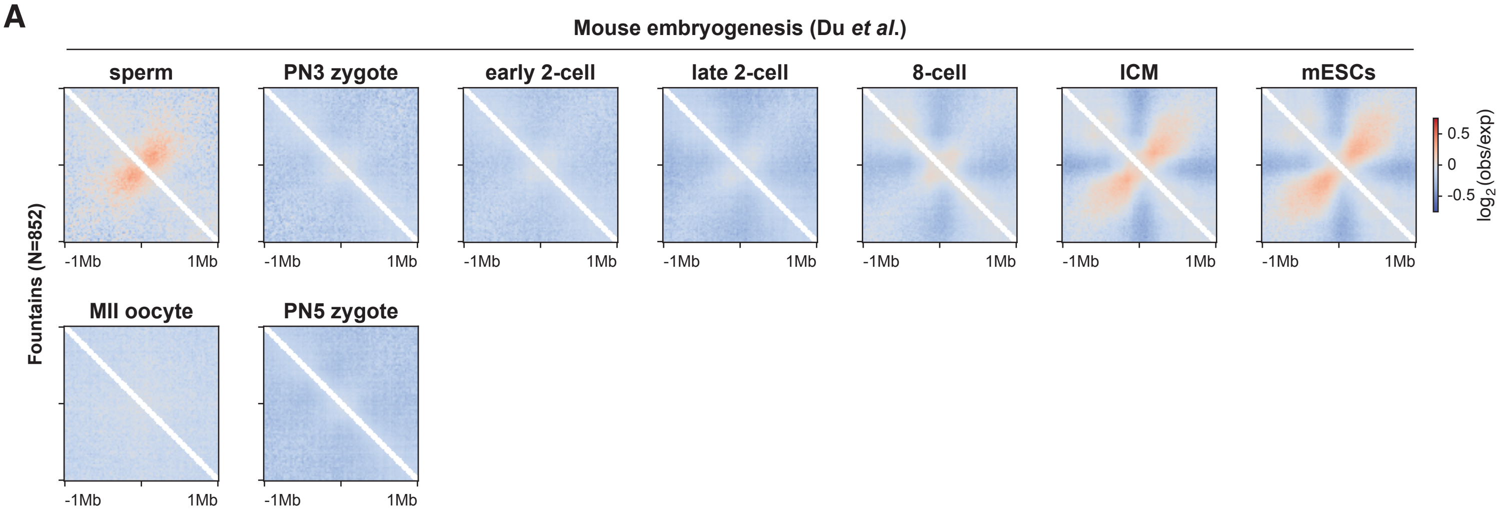
Mouse embryogenesis (Du et al.)
sperm
PN3 zygote
early 2-cell
late 2-cell
8-cell
ICM
mESCs
MII oocyte
PN5 zygote
Fountains (N=852)
-1Mb
1Mb
0.5
0
-0.5
log2(obs/exp)

B

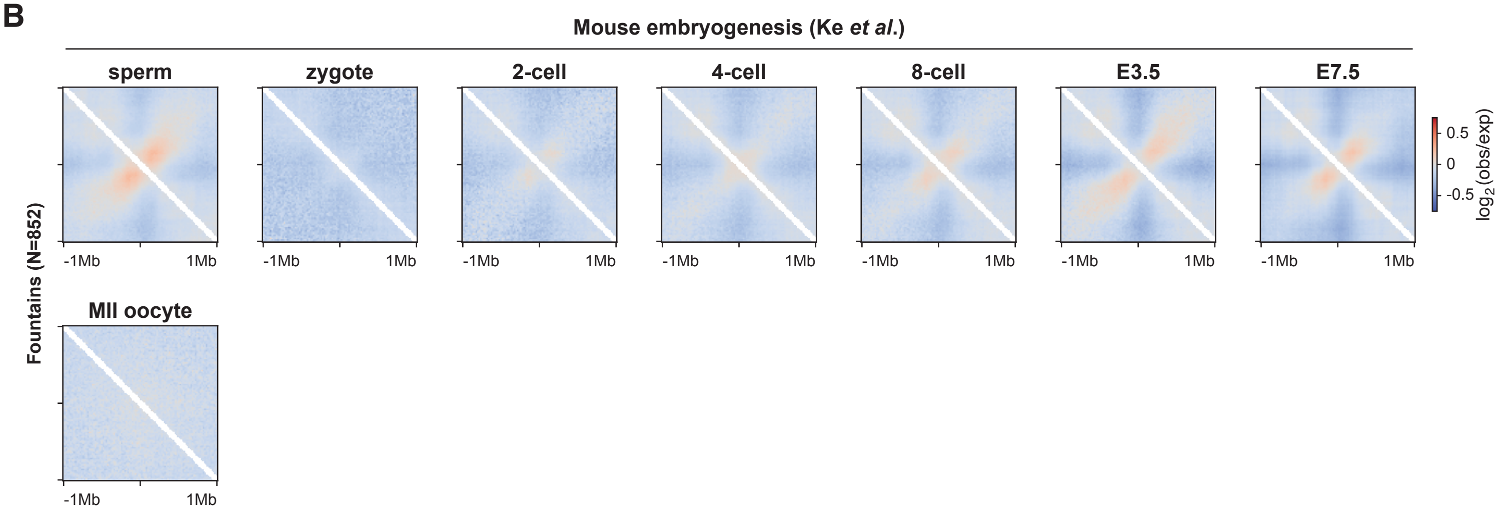
Mouse embryogenesis (Ke et al.)
sperm
zygote
2-cell
4-cell
8-cell
E3.5
E7.5
MII oocyte
Fountains (N=852)
-1Mb
1Mb
0.5
0
-0.5
log2(obs/exp)

Figure S6. Fountains are established during early mouse development. (A, B) Aggregate region analysis of the fountains in publicly available mouse early development datasets (5,6) showing gradual establishment of fountains following the developmental stage progression. The samples are sorted from left to right by their developmental stage. Annotation of fountains is taken from WAPL/CTCF co-depleted cells.

Figure S7

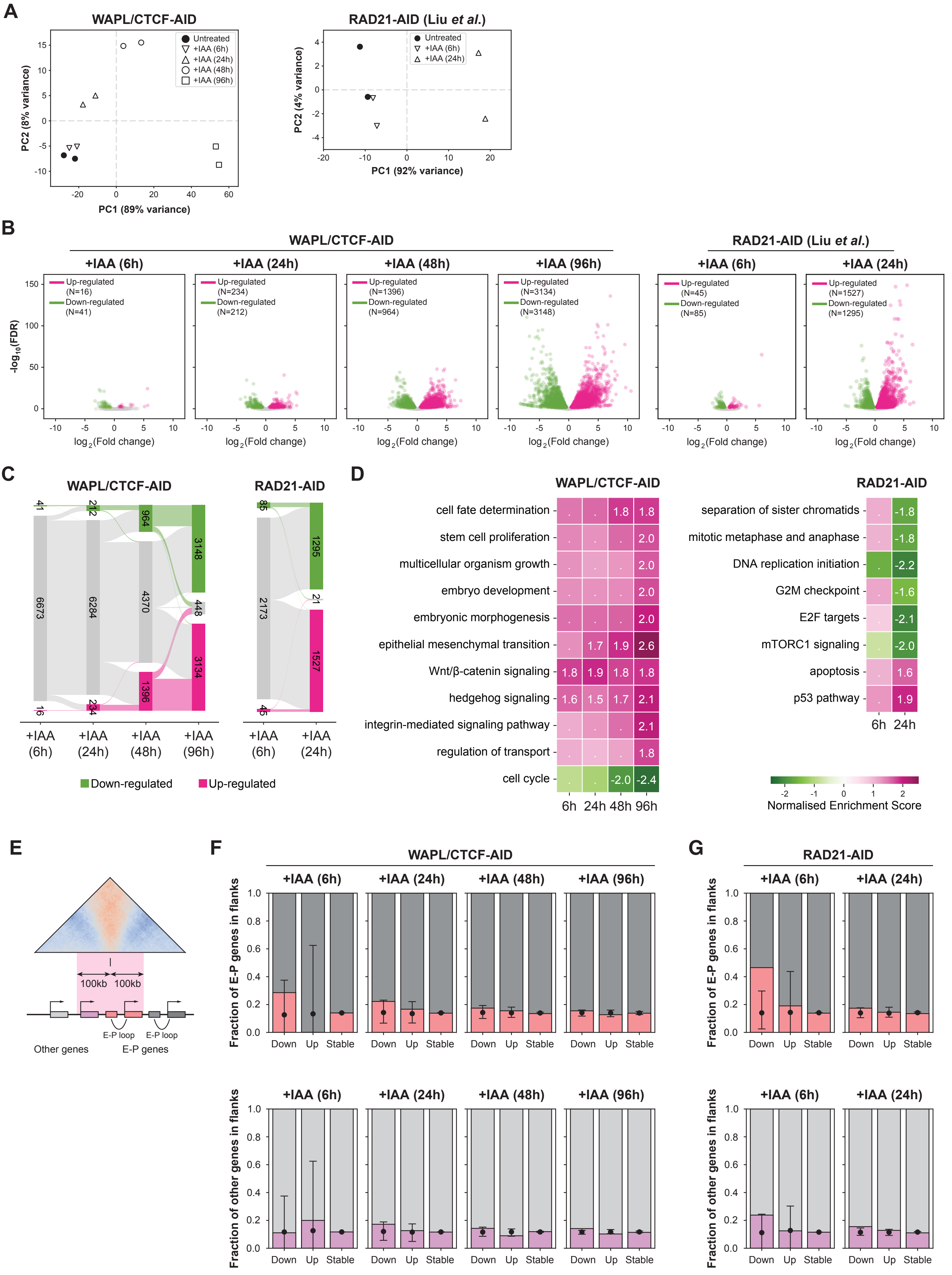

A
WAPL/CTCF-AID
Untreated
+IAA (6h)
+IAA (24h)
+IAA (48h)
+IAA (96h)
PC2 (8% variance)
PC1 (89% variance)
RAD21-AID (Liu et al.)
PC2 (4% variance)
PC1 (92% variance)
B
WAPL/CTCF-AID
RAD21-AID (Liu et al.)
+IAA (6h)
Up-regulated (N=16)
Down-regulated (N=41)
+IAA (24h)
Up-regulated (N=234)
Down-regulated (N=212)
+IAA (48h)
Up-regulated (N=1396)
Down-regulated (N=964)
+IAA (96h)
Up-regulated (N=3134)
Down-regulated (N=3148)
+IAA (6h)
Up-regulated (N=45)
Down-regulated (N=85)
+IAA (24h)
Up-regulated (N=1527)
Down-regulated (N=1295)
-log10(FDR)
log2(Fold change)
C
WAPL/CTCF-AID
RAD21-AID
+IAA (6h)
+IAA (24h)
+IAA (48h)
+IAA (96h)
Down-regulated
Up-regulated
D
WAPL/CTCF-AID
cell fate determination
stem cell proliferation
multicellular organism growth
embryo development
embryonic morphogenesis
epithelial mesenchymal transition
Wnt/β-catenin signaling
hedgehog signaling
integrin-mediated signaling pathway
regulation of transport
cell cycle
6h 24h 48h 96h
RAD21-AID
separation of sister chromatids
mitotic metaphase and anaphase
DNA replication initiation
G2M checkpoint
E2F targets
mTORC1 signaling
apoptosis
p53 pathway
6h 24h
Normalised Enrichment Score
E
100kb 100kb
E-P loop
E-P loop
Other genes
E-P genes
F
WAPL/CTCF-AID
G
RAD21-AID
+IAA (6h)
+IAA (24h)
+IAA (48h)
+IAA (96h)
Fraction of E-P genes in flanks
Fraction of other genes in flanks
Down
Up
Stable

Figure S7. RNA-seq analysis following WAPL/CTCF and RAD21 depletions. (A) Principal component analysis of the RNA-seq data in WAPL/CTCF (left panel) and RAD21 (right panel) depleted mESCs. **(B)** Volcano plot showing effect sizes and significance of the down-regulated (green) and upregulated (pink) genes measured by RNA-seq after 6, 24, 48, and 96 hours of WAPL/CTCF (left panel) and 6 and 24 hours of RAD21 (right panel) depletion. The number of differentially expressed genes at each time point is indicated in the top right corners (FDR < 0.01). **(C)** Sankey diagram showing the up-regulated (pink) and down-regulated (green) genes following WAPL/CTCF (left panel) and RAD21 (right panel) depletion. The data for the RAD21-AID cell line is from (7). **(D)** Gene set enrichment analysis of RNA-seq data after WAPL/CTCF (left panel) and RAD21 (right panel) depletion (up-regulated gene sets in pink, down-regulated gene sets in green). The normalised enrichment score for significant gene sets (FDR < 0.1) is shown. **(E)** Schematic for the intersection of the genes with the 100 kb fountain bases flanks. Genes were classified as EP genes if their TSS was located within 2kb from the annotated E-P loops (8). The 100 kb flanks of fountain bases were intersected with expressed genes in WAPL/CTCF-AID or RAD21-AID cell lines. Fractions of differentially expressed EP genes (top) and other genes (bottom) found in the fountain bases flanks (in coral and purple) and in the rest of the genome (in grey) after WAPL/CTCF **(F)** and RAD21 **(G)** depletion. Four gene categories used for the analysis are represented in different colors, matching the ones on the schematic. Mean fraction of genes obtained through label permutations is shown as a black dot. Whiskers represent the maximum and minimum values obtained in permutations.

References

1. Busslinger, G.A., Stocsits, R.R., van der Lelij, P., Axelsson, E., Tedeschi, A., Galjart, N. and Peters, J.M. (2017) Cohesin is positioned in mammalian genomes by transcription, CTCF and Wapl. *Nature*, **544**, 503-507.
2. Bonev, B., Mendelson Cohen, N., Szabo, Q., Fritsch, L., Papadopoulos, G.L., Lubling, Y., Xu, X., Lv, X., Hugnot, J.P., Tanay, A. *et al.* (2017) Multiscale 3D Genome Rewiring during Mouse Neural Development. *Cell*, **171**, 557-572 e524.
3. Nora, E.P., Goloborodko, A., Valton, A.L., Gibcus, J.H., Uebersohn, A., Abdennur, N., Dekker, J., Mirny, L.A. and Bruneau, B.G. (2017) Targeted Degradation of CTCF Decouples Local Insulation of Chromosome Domains from Genomic Compartmentalization. *Cell*, **169**, 930-944 e922.
4. Nora, E.P., Caccianini, L., Fudenberg, G., So, K., Kameswaran, V., Nagle, A., Uebersohn, A., Hajj, B., Saux, A.L., Coulon, A. *et al.* (2020) Molecular basis of CTCF binding polarity in genome folding. *Nat Commun*, **11**, 5612.
5. Du, Z., Zheng, H., Huang, B., Ma, R., Wu, J., Zhang, X., He, J., Xiang, Y., Wang, Q., Li, Y. *et al.* (2017) Allelic reprogramming of 3D chromatin architecture during early mammalian development. *Nature*, **547**, 232-235.
6. Ke, Y., Xu, Y., Chen, X., Feng, S., Liu, Z., Sun, Y., Yao, X., Li, F., Zhu, W., Gao, L. *et al.* (2017) 3D Chromatin Structures of Mature Gametes and Structural Reprogramming during Mammalian Embryogenesis. *Cell*, **170**, 367-381 e320.
7. Liu, N.Q., Maresca, M., van den Brand, T., Braccioli, L., Schijns, M., Teunissen, H., Bruneau, B.G., Nora, E.P. and de Wit, E. (2021) WAPL maintains a cohesin loading cycle to preserve cell-type-specific distal gene regulation. *Nat Genet*, **53**, 100-109.
8. Hsieh, T.S., Cattoglio, C., Slobodyanyuk, E., Hansen, A.S., Darzacq, X. and Tjian, R. (2022) Enhancer-promoter interactions and transcription are largely maintained upon acute loss of CTCF, cohesin, WAPL or YY1. *Nat Genet*, **54**, 1919-1932.
